# Transformed notochordal cells trigger chronic wounds in zebrafish, destabilizing the vertebral column and bone homeostasis

**DOI:** 10.1242/dmm.047001

**Published:** 2021-03-19

**Authors:** Paco López-Cuevas, Luke Deane, Yushi Yang, Chrissy L. Hammond, Erika Kague

**Affiliations:** 1 School of Biochemistry, Biomedical Sciences Building, University of Bristol, Bristol BS8 1TD, UK; 2 School of Physics, HH Wills Physics Laboratory, University of Bristol, Bristol BS8 1TL, UK; 3 Centre for Nanoscience and Quantum Information, University of Bristol, Bristol BS8 1FD, UK; 4 Bristol Centre for Functional Nanomaterials, University of Bristol, Bristol BS8 1TL, UK; 5 The School of Physiology, Pharmacology and Neuroscience, Biomedical Sciences, University of Bristol, Bristol BS8 1TD, UK

**Keywords:** Chordoma, Notochord, Vertebral column, Zebrafish intervertebral disc, Inflammation, Bone homeostasis

## Abstract

Notochordal cells play a pivotal role in vertebral column patterning, contributing to the formation of the inner architecture of intervertebral discs (IVDs). Their disappearance during development has been associated with reduced repair capacity and IVD degeneration. Notochord cells can give rise to chordomas, a highly invasive bone cancer associated with late diagnosis. Understanding the impact of neoplastic cells during development and on the surrounding vertebral column could open avenues for earlier intervention and therapeutics. We investigated the impact of transformed notochord cells in the zebrafish skeleton using a line expressing RAS in the notochord under the control of the *kita* promoter, with the advantage of adulthood endurance. Transformed cells caused damage in the notochord and destabilised the sheath layer, triggering a wound repair mechanism, with enrolment of sheath cells (*col9a2^+^*) and expression of *wt1b*, similar to induced notochord wounds. Moreover, increased recruitment of neutrophils and macrophages, displaying abnormal behaviour in proximity to the notochord sheath and transformed cells, supported parallels between chordomas, wound and inflammation. Cancerous notochordal cells interfere with differentiation of sheath cells to form chordacentra domains, leading to fusions and vertebral clefts during development. Adults displayed IVD irregularities reminiscent of degeneration, including reduced bone mineral density and increased osteoclast activity, along with disorganised osteoblasts and collagen, indicating impaired bone homeostasis. By depleting inflammatory cells, we abrogated chordoma development and rescued the skeletal features of the vertebral column. Therefore, we showed that transformed notochord cells alter the skeleton during life, causing a wound-like phenotype and activating chronic wound response, suggesting parallels between chordoma, wound, IVD degeneration and inflammation, highlighting inflammation as a promising target for future therapeutics.

This article has an associated First Person interview with the first author of the paper.

## Abstract (precis)

**Summary:** Analyses using a zebrafish line expressing RAS in the notochord, under the control of the *kita* promoter, revealed that transformed notochord cells alter the skeleton during life, causing a wound-like phenotype and activating chronic wound response.

## INTRODUCTION

The vertebral column is the central axis of the skeleton in all vertebrates. It is composed of segments (vertebrae) connected by joint-like structures called intervertebral discs (IVDs). In mammals, the architecture of the IVDs is made by an annulus fibrosus (AF), a collagenous layer surrounding a hydrated and gelatinous nucleus pulposus (NP) core, which contains chondrocyte-like cells derived from embryonic notochord cells (Rodrigues-Pinto et al., 2014). The disappearance of notochordal cells in mammals during development of the vertebral column has been linked to reduction of repair capacity and IVD degeneration (IVDD) (Wang et al., 2017). Occasionally, notochordal cells can cause vertebral malformations, and, in rare cases, cell transformation leads to chordomas (Salisbury, 1993; Choi et al., 2008), a rare bone cancer of the axial skeleton and skull base (McMaster et al., 2011).

With an incidence of approximately one in a million, chordomas account for ∼1-4% of all primary bone malignancies and 20% of primary spinal tumours (Chugh et al., 2007). Chordomas are slow growing and highly resistant to both chemotherapy and radiotherapy, meaning that radical surgery is often the primary choice for treatment modality (McMaster et al., 2011). Unfortunately, in many cases, the proximity of chordomas to vital structures means that local excision is rarely achieved, resulting in a recurrence rate greater than 50% (Stacchiotti et al., 2017; Barry et al., 2011). Distant metastases to lung, bone, soft tissue, lymph node, liver and skin have been reported in up to 43% of cases (Stacchiotti et al., 2017; Barry et al., 2011). Interestingly, chordomas lead to changes in bone quality, and often appear on X-rays and computerised tomography (CT) as eroding bone lesions with associated soft-tissue calcification (de Bruine and Kroon, 1988), suggesting that modifications in the behaviour of the NP cells disrupt disc and bone homeostasis. Impairment of disc homeostasis is a hallmark of IVDD (Novais et al., 2020b), which, unlike chordomas, is very common, representing the most common cause of back pain (Zheng and Chen, 2015), a symptom that 80% of the adult world population suffer from (GBD 2016 Disease and Injury Incidence and Prevalence Collaborators, 2017). How transformed NP cells affect the IVD and surrounding vertebrae during their development is currently unknown, and no animal models to show how transformed cells dynamically interact with and affect the IVDs and vertebral column *in vivo* have been described. Such models could contribute to our understanding of chordoma development, IVDD, the interaction of the NP with the skeletal tissues and possible therapeutic avenues for both conditions.

Zebrafish have emerged as an advantageous animal model for a variety of human diseases, including cancer and skeletal diseases, owing to their fast development, tractability, flexible genetic manipulation (transgenesis, forward and reverse genetics) and translucency (Bergen et al., 2019). Reporter lines allow *in vivo* assessment of cell behaviour not only during early development but also during the later stages of skeletal formation in juveniles (Bergen et al., 2019). Zebrafish have high tissue regenerative capacity, with the ability to restore vacuolated cells of the notochord upon injury (Garcia et al., 2017). In zebrafish, notochord cells remain throughout life; they are enveloped by a sheath layer that acts as a sealing basement membrane to isolate the inner notochord vacuolated cells and carries high potential to mineralise (Fleming et al., 2004; Stemple, 2005). The notochord sheath plays an important role in the segmentation of the vertebral column and centra primordium (chordacentra) formation (Lleras Forero et al., 2018; Pogoda et al., 2018; Wopat et al., 2018). Following genetic manipulation, mechanical injury (needle punctures) or chemical treatment (with nystatin), repair of tissue damage appears to involve a subpopulation of notochord sheath cells that become activated, expressing *Wilms tumor 1b* (*wt1b*), and migrate towards the wound, setting landmarks during notochord repair (Garcia et al., 2017; Lopez-Baez et al., 2018).

Chordoma onset has been described in larval zebrafish expressing the oncogene *RAS* in the notochord, using the bimodal Gal4/UAS system and activation of the oncogenic RTK/Ras pathway (Burger et al., 2014). These zebrafish chordoma models become affected within the first 3 days post-fertilisation (dpf), progressively developing notochord hyperplasia, similar to histological features of human chordomas (Burger et al., 2014). Recently, the zebrafish chordoma model was used to test genetic potential to transform the notochord *in vivo*, providing suggestive evidence that Brachyury (*TBXT*), a highly expressed gene in human chordomas (Vujovic et al., 2006), is insufficient to initiate chordomas, instead suggesting activation of members of the RTK signalling pathway as potential players in chordoma formation (D'Agati et al., 2019). The behaviour of notochord cancer cells during zebrafish life has not yet been studied, owing to early lethality of chordoma models during larval stages. It is unknown whether notochord cancer cells trigger a wound repair mechanism similar to those of notochord injury models, which activate an acute inflammatory response as is seen in other early cancers (Feng et al., 2012, 2010). It is also unclear whether notochord cancer cells exert control as notochordal remnants to interfere with bone formation and, later in life, with bone homeostasis.

Here, we studied the interactions between the notochord cancer cells within the forming vertebral column and bone homeostasis using a well-characterised transgenic line, *kita-RAS*, which drives expression of HRASV12 in the notochord (and in melanoblasts, thus modelling melanoma) and survives to adulthood (Santoriello et al., 2010; van den Berg et al., 2019; Feng et al., 2012). We showed that ‘transformed’ notochord cells destabilise the notochord sheath layer, activating a chronic wound repair response similar to those caused by induced notochord wounds previously described (Garcia et al., 2017; Lopez-Baez et al., 2018). These pre-neoplastic cells lead to invagination of the *col9a2*-expressing notochord sheath cells towards the wound and participation of *wt1b* notochord sheath subpopulation. Interestingly, macrophages and neutrophils were present in higher numbers and showed prolonged interaction with the wounded notochord sheath layer, as described in other cancers. The metameric pattern of segmentation of the vertebral column was compromised, but not abrogated, leading to vertebral fusions and clefts. Adult bone homeostasis was altered, as observed by differences in vertebral bone mineral density and collagen fibre distribution. Transformed cells also compromised the adult zebrafish equivalent IVD architecture, leading to NP ‘scar’ tissue, NP cellular disorganisation and affecting the structure of the AF, similar to IVDD. Chordoma development and skeletal defects were rescued when we partially depleted neutrophils and macrophages. In conclusion, our results indicate that transformed notochord cells cause chronic wounds, leading to inflammation, vertebral abnormalities, and disc and bone homeostasis impairment. Chordoma development could be controlled by limiting inflammation, revealing new avenues for therapeutics and highlighting the use of zebrafish as an animal model.

## RESULTS

### *kita-RAS* induces wound-like destabilisation of the notochord

**Fig. 1.**
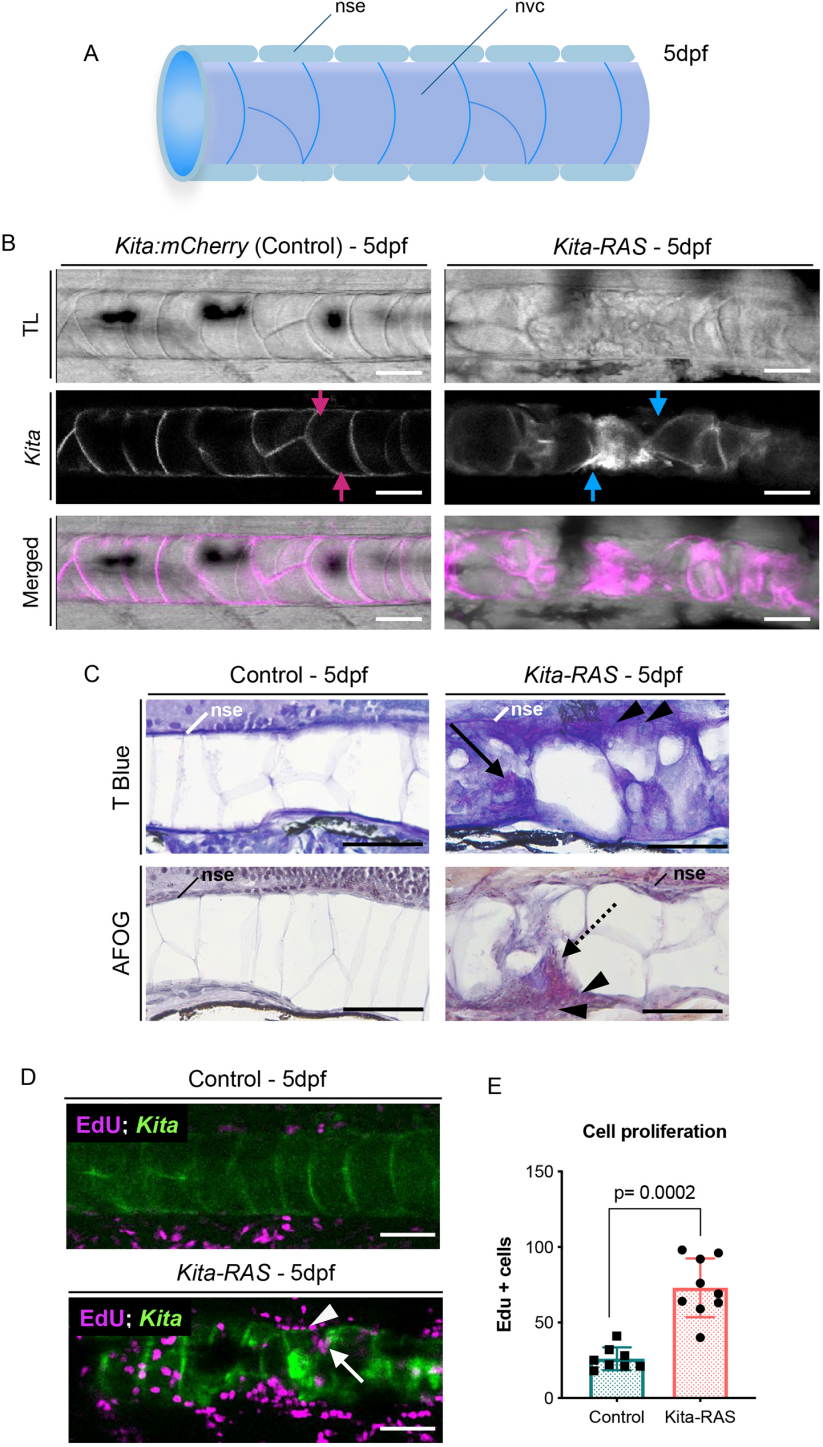
***kita* drives RAS expression in the notochord,** **inducing fibrosis and wound-like phenotype.** (A) Schematic of the wild-type notochord. The notochord is a rod tube formed by a sealing notochord sheath epithelium (nse) layer that wraps the notochord vacuolated cells (nvc). dpf, days post-fertilisation. (B) Maximum projections from confocal images of control (*kita:mCherry*) and *kita-RAS-mCherry* (*kita-RAS*) at 5 dpf. *kita* drives expression of the reporter and RAS in the notochord cells (magenta arrows), leading to dramatic changes in the notochord. Gaps between vacuolated notochord cells (blue arrows) are filled with small non-vacuolated cells. (C) Histological sections of 5 dpf control (*kita-mCherry*) and *kita-RAS* larvae, stained with Toluidine Blue (T Blue) and AFOG. Control fish show an intact nse. *kita-RAS* larvae show disruptions of the nse (arrowheads), accumulation of non-vacuolated cells within the notochord (arrow) and fibrous tissue (AFOG, dashed line arrow). (D) Cross section from confocal images of *kita* (control) and *kita-RAS* at 5 dpf, treated with EdU from 2 dpf to 4 dpf to show cell proliferation. Note increased proliferation in the notochord sheath (arrowhead) and within wounded areas of the notochord (arrow). (E) Quantification of cell proliferation was performed by counting the number of EdU^+^ cells in the control (*n*=8) and *kita-RAS* (*n*=9). Nonparametric *t*-test, post hoc Mann–Whitney test; data are mean±s.d. Scale bars: 50 µm.

Notochord-specific *Gal4* lines crossed to *UAS:EGFP-HRASV12* have been previously described as powerful models for inducing chordomas in zebrafish (Burger et al., 2014). A transgenic line extensively used to induce melanoma, in which *HRASV12* expression is driven by the *kita* promoter in melanoblasts, goblet cells and notochord cells [owing to the presence of an enhancer element for *tiggy winkle hedgehog* (*twhh*)] (Distel et al., 2009) has the advantage over other notochord RAS-expressing lines because it survives to adulthood (Santoriello et al., 2010; van den Berg et al., 2019). We used *kita-RAS-GFP* and *kita-RAS-mCherry* to study the progressive changes of the transformed notochord cells and their interaction with the forming vertebral column. In 5 dpf zebrafish larvae, the outer layer of the notochord is formed by an epithelial-like sheath wrapping notochord vacuolated cells (Wopat et al., 2018) (Fig. 1A). Confocal images through the notochord, at 5 dpf, showed that *kita* drives reporter expression in the notochord vacuolated cells, but not in the sheath cells (Fig. 1B). As in other chordoma RAS models, *kita-RAS* led to dramatic destabilisation of the notochord vacuolated cells starting as early as 3 dpf and by 5 dpf affected 70% of the larvae (>200 larvae analysed). Affected larvae were considered when they displayed more than three lesions in the notochord. Each lesion was characterised by increased RAS expression and abnormal notochord cell morphology (Fig. 1B). At the same developmental stage (5 dpf), notochord cells were interspaced by infiltration of non-vacuolated cells and accumulation of fibrous collagenous tissue [Acid Fuchsin Orange G (AFOG) staining, red colour] (Fig. 1B,C). Furthermore, histological sections suggested local destabilisation of the notochord sheath layer at the region of collapsed vacuolated cells (Fig. 1C). To analyse cell proliferation, we treated larvae with 5-ethynyl-2′-deoxyuridine (EdU) solution to be incorporated into the DNA of proliferating cells from 2 dpf to 4 dpf and counted the number of EdU^+^ cells at 5 dpf from confocal images. Notochord cells and notochord sheath cells in *kita-RAS* are highly proliferative (*P*=0.0002) (Fig. 1D,E). Interestingly, the organisation of the notochord in *kita-RAS* fish displayed cellular characteristics reminiscent of those observed in notochord wounding models (needle puncture) (Lopez-Baez et al., 2018) (Fig. S1), suggesting that chordoma may recapitulate repair mechanisms, as has been suggested for several other cancers (Feng et al., 2010).

### Pre-neoplastic notochord cells trigger the notochord wound repair mechanism in zebrafish

**Fig. 2.**
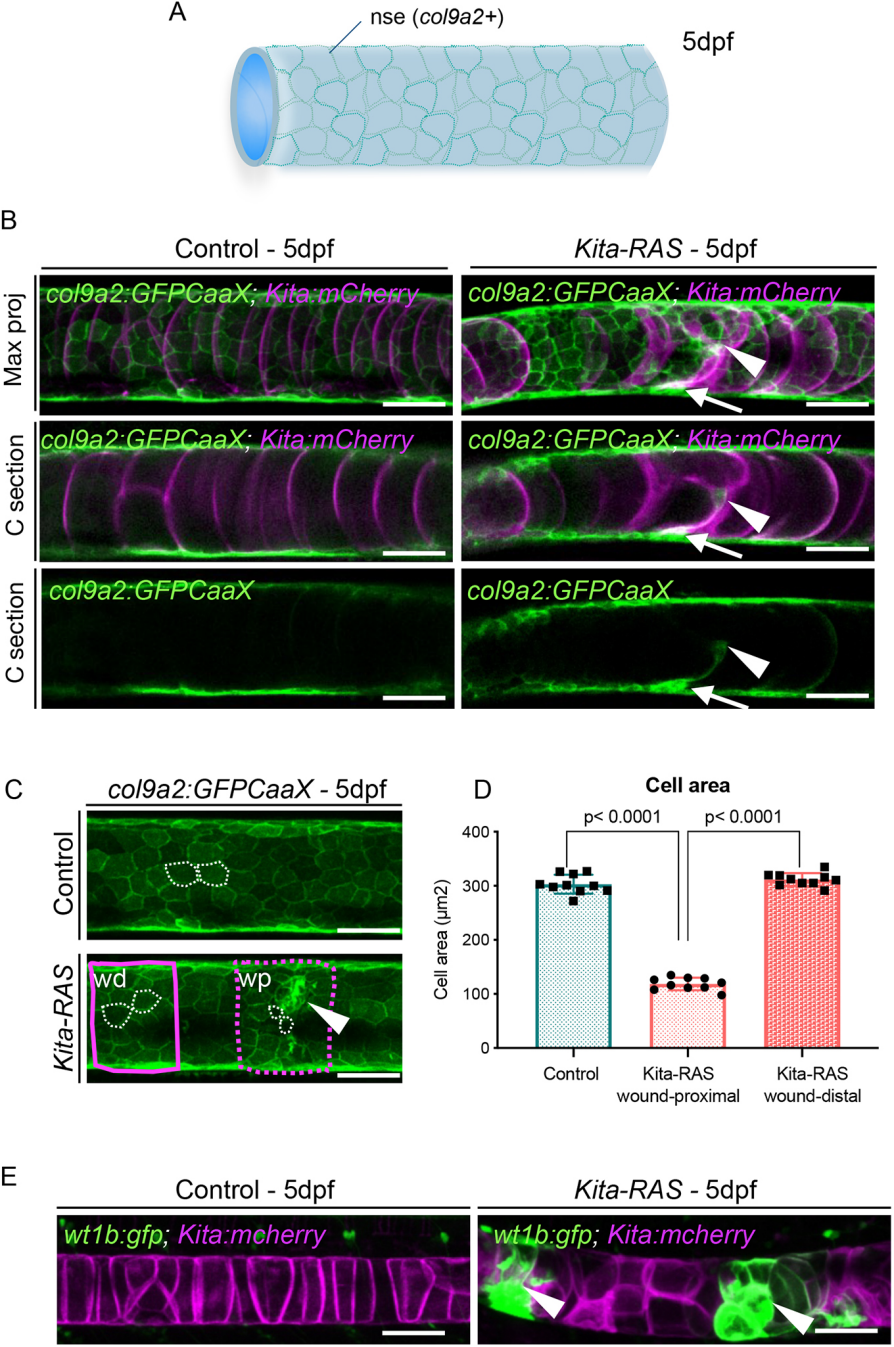
**Transformed notochord cells alter the organisation of the sheath layer and activate wound repair mechanisms.** (A) Schematic of the notochord and notochord sheath epithelium (nse) at 5 dpf, formed by cells that highly express collagen type IX. (B) Confocal images showing maximum projections (Max proj) and cross sections (C section) of *col9a2:GFPCaaX* (nse) and *kita-mCherry* (notochord cells) in control and *kita-RAS*, at 5 dpf. In *kita-RAS*, a ‘scar’ region within the notochord (arrowheads) expresses *col9a2*, and shows connectivity with the nse (arrows). (C) The areas (white dashed lines) of notochord sheath cells were analysed in controls and within two regions of *kita-RAS* expressing *col9a2:GFPCaaX:* proximal (wp; magenta dashed line) and distal (wd; magenta solid line) to the wound (arrowhead). (D) Cell area quantification of each group (ten cells were measured for each group and region, and *n*=10 fish per group). Nested one-way ANOVA and Tukey's multiple comparisons test were used for statistical analysis. Data are mean±s.d. *P*-values are indicated when significant (*P*<0.05). (E) Maximum projections from confocal images showing expression of *wt1b:gfp* in the wounded regions (arrowheads) of *kita-RAS*. *w1b* is not expressed in controls. Scale bars: 50 µm.

Wounds in the notochord induced by needle injury, amputation and chemical damage lead to the collapse of notochord vacuolated cells, sheath cell invasion and expression of *wt1b* within a cell subpopulation of the notochord sheath (Garcia et al., 2017; Lopez-Baez et al., 2018). To investigate whether pre-neoplastic notochord cells mimic a wound-like response, we crossed *kita-RAS-mCherry* with Tg(*col9a2:GFPCaaX*), a marker for the notochord sheath layer (Fig. 2A), and with Tg(*wt1b:gfp*), to label the subpopulation of sheath cells that standardly respond to damage. We confirmed that, at 5 dpf, *kita* is not expressed in the notochord sheath layer, and only in vacuolated notochord cells (Fig. 2B). We observed *col9a2* expression in regions of damage within the notochord, suggesting sheath cell migration towards the chordoma wounded area (Fig. 2B). Cross sections through the notochord, at 5 dpf, showed *col9a2*-expressing cells within the notochord in connection with the notochord sheath (Fig. 2B), reinforcing the possible migration of sheath cells to the lesioned region. To check for cell abnormalities in the notochord sheath, we quantified the cell area of the *col9a2^+^* cells of severely affected larvae within two regions of our *kita-RAS* wound: proximal and distal (Fig. 2C). *kita-RAS* showed significant reduction in cell area in wound-proximal regions (*P*<0.0001), but not in wound-distal regions, compared to controls (Fig. 2D). We did not detect cell area changes in the sheath layer of less affected larvae. Therefore, wound-like lesions caused by transformed notochord cells led to local cellular modifications in the sheath layer. Next, we analysed *wt1b* expression in the *kita-RAS* outcrossed fish. Control fish exhibited no expression of *wt1b* in the notochord, whereas *kita-RAS* showed strong *wt1b* expression by pre-neoplastic cells located at severe wounded regions in 100% of the cases analysed (20/20) (Fig. 2E). These findings corroborate strong parallels between cancer and wound repair (MacCarthy-Morrogh and Martin, 2020).

### Wounded notochord sheath elicits prolonged recruitment of innate inflammatory cells

**Fig. 3.**
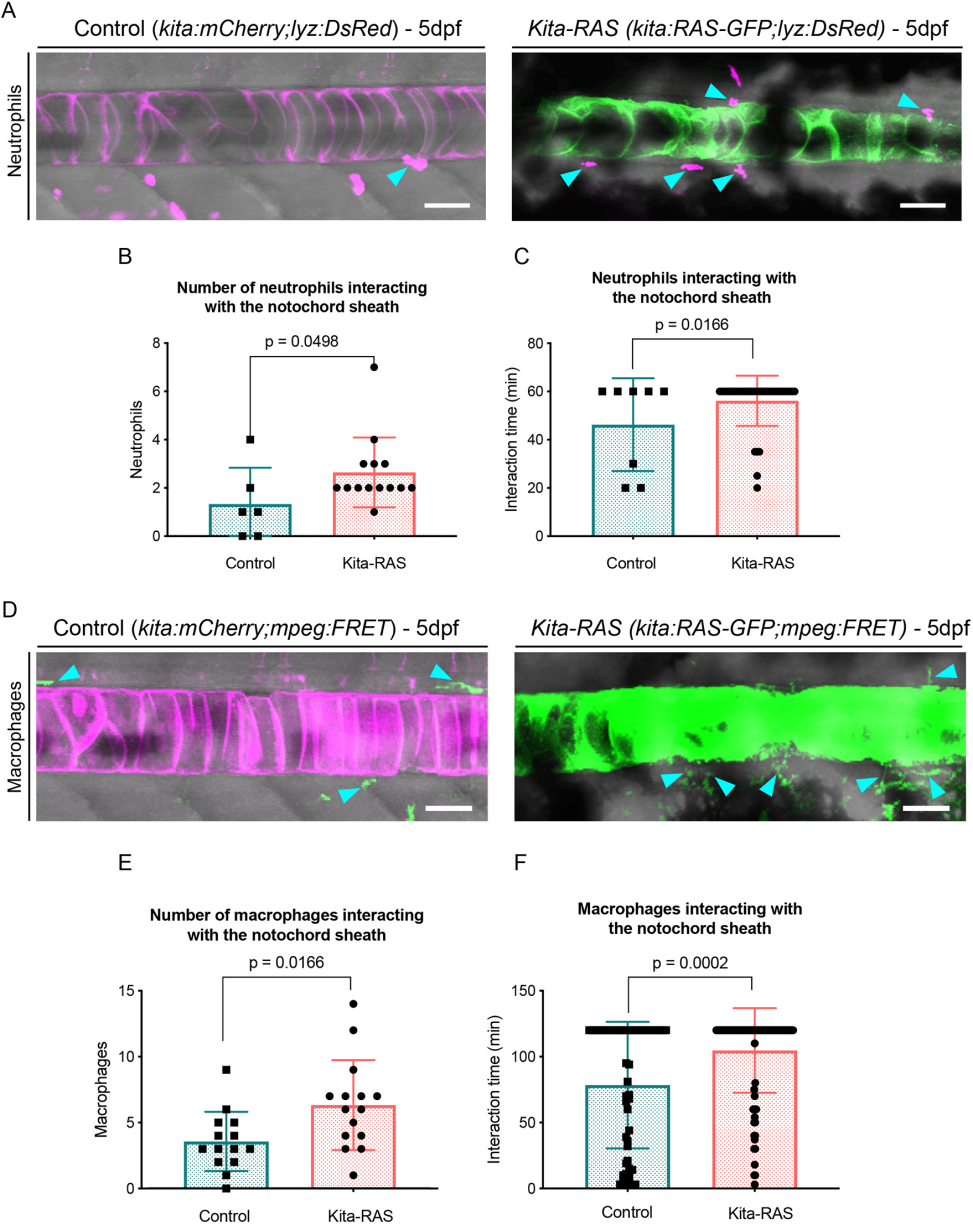
**Increased inflammatory response detected in the notochord sheath of *kita-RAS*.** (A) Maximum projections from confocal images of the notochord at 5 dpf in control (*kita-mCherry*) and *kita-RAS*, showing neutrophils (cyan arrowheads) interacting by contact with the notochord sheath layer. (B) Numbers of neutrophils interacting with the notochord sheath during the time lapse (controls *n*=6 fish, *kita-RAS n*=14 fish). (C) Interaction time between neutrophils and the notochord sheath during the time-lapse movies. Each dot or square represents one neutrophil (controls *n*=8 neutrophils, *n*=4 fish; *kita-RAS n*=39 neutrophils, *n*=14 fish). (D) Maximum projections from confocal images of the notochord of 5 dpf control (*kita-mCherry*) and *kita-RAS* fish, showing macrophages (cyan arrowheads) interacting by contact with the notochord sheath. (E) Numbers of macrophages interacting with the notochord sheath during the time lapse (controls *n*=14 fish, *kita-RAS n*=15 fish). (F) Interaction time between macrophages and the notochord sheath during the time-lapse movies. Each dot or square represents one macrophage (controls *n*=51 macrophages, *n*=13 fish; *kita-RAS n*=95 macrophages, *n*=15 fish). Unpaired, nonparametric *t*-test and Mann–Whitney test were used for all charts. Data are mean±s.d.; *P*-values are indicated when significant (*P*<0.05). Scale bars: 50 µm.

Several studies have reported that oncogene-transformed cells trigger an innate inflammatory response, with both neutrophils and macrophages recruited to the pre-cancerous tissue (Chia et al., 2018; Feng et al., 2010; Freisinger and Huttenlocher, 2014; Roh-Johnson et al., 2017). This recruitment of neutrophils and macrophages is responsible for clearing cell debris and orchestrates tissue repair responses including wound angiogenesis and matrix deposition (Eming et al., 2017). We questioned whether oncogenic RAS expression in the notochord cells and the lesioned notochord sheath might also induce an inflammatory response in our zebrafish chordoma model. During the first weeks of development, zebrafish do not have a functional adaptive immune system, allowing us to investigate the innate immune response on its own (Renshaw and Trede, 2012). We performed time-lapse imaging at 5 dpf and analysed the interactions of neutrophils and macrophages with the notochord sheath layer. For neutrophils, we incrossed Tg(*kita:Gal4;UAS:mCherry;UAS:HRASG12V-GFP;lyz:DsRed*), and selected RAS^–^/Lyz^+^ larvae, Tg(*kita:mCherry;lyz:DsRed*), and RAS^+^/Lyz^+^ larvae, Tg(*kita:HRASG12V-GFP;lyz:DsRed*), as controls and *kita-RAS* fish, respectively. For macrophages, we incrossed Tg(*kita:Gal4;UAS:mCherry;UAS:HRASG12V-GFP;mpeg:FRET*) and selected RAS^–^/Mpeg^+^ larvae, Tg(*kita:mCherry;mpeg:FRET*), and RAS^+^/Mpeg^+^ larvae, Tg(*kita:HRASG12V-GFP;mpeg:FRET*), as controls and *kita-RAS* fish, respectively. Higher numbers of neutrophils and macrophages were recruited, making a prolonged direct contact with the wounded notochord sheath in *kita-RAS* in comparison to controls (Fig. 3; Fig. S2, Movies 1 and 2), similarly to the inflammatory response previously reported in the melanoma model (Feng et al., 2010). Remarkably, we also found neutrophils and macrophages infiltrating wounded regions and in direct contact with notochord vacuolated cells (Fig. S2, Movies 1 and 2). Together, our results showed that zebrafish chordoma induces a chronic notochord inflammatory wound response with typical wound recruitment of neutrophils and macrophages. Inflammatory cells trespass the notochord sheath layer in wounded regions to form direct contact with transformed notochord cells, a similar behaviour to that described for other cancers (Feng et al., 2012).

### Depletion of neutrophils and macrophages abolishes chordoma development

**Fig. 4.**
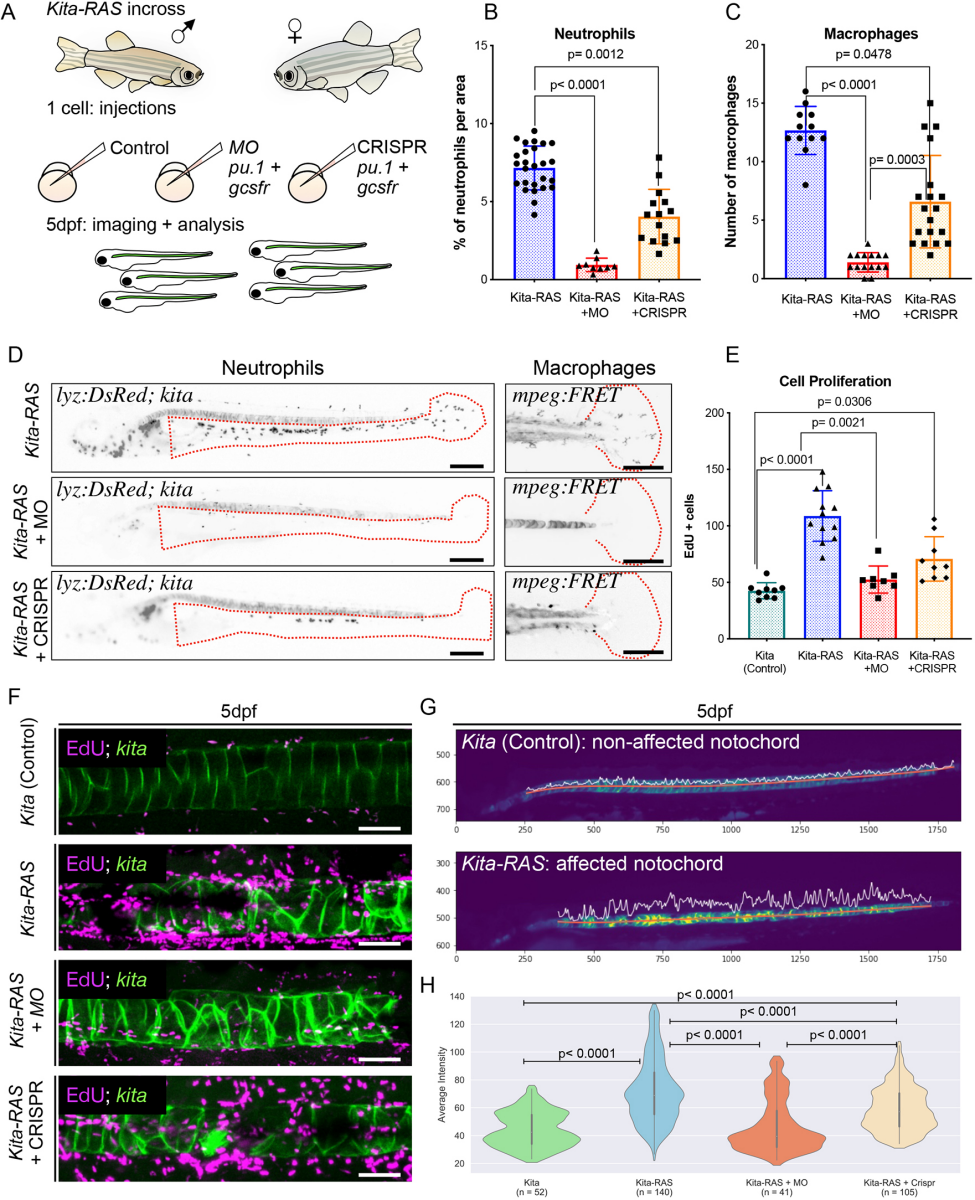
**Modulation of the innate immune response prevents chordoma.** (A) Schematics of the experiment. *kita-RAS-GFP* were incrossed, and embryos from the same cross were divided into three groups: controls, morpholinos (MO) or CRISPR targeting *pu.1+gcsfr* (for depletion of neutrophils and macrophages). Injections were carried out at one-cell stage. The notochords were subsequently imaged and analysed at 5 dpf. (B) Percentage of neutrophils per area in *kita-RAS* (control group *n*=26) and *kita-RAS* injected with either MO (*n*=9) or CRISPR (*n*=15). (C) Numbers of macrophages in *kita-RAS* (*n*=14) and *kita-RAS* injected with either MO (*n*=15) or CRISPR (*n*=19). (D) For quantification of neutrophils and macrophages, injections were carried out in Tg(*lyz:DsRed;mpeg:FRET:kita:mCherry*). Percentage of neutrophils was calculated within the selected area (regions within the red dashed lines), after image binarisation. Numbers of macrophages were manually counted in the dorsal fin area (regions within the red dashed lines). Images are displayed with inverted colour and in black and white for better visualisation. Scale bars: 250 µm. (E) Cell proliferation was quantified from confocal images, by counting numbers of EdU^+^ cells in *kita* (control) (*n*=9), *kita-RAS* (control for injections) (*n*=12) and *kita-RAS* injected with MO (*n*=8) or CRISPR (*n*=9). (F) Maximum projections from confocal images to show cell proliferation in each of the experimental groups. Scale bars: 50 µm. (G) Computational analysis was performed on images acquired under a stereomicroscope at 5 dpf, and was based on the intensity profile derived from the fluorescence of the identified notochord (red lines). Peaks along the notochord represent the intensity profile. Lesions are identified by higher pixel intensity and broader area under the peak. *x*- and *y*-axes show numbers of pixels and serve as scale bars. (H) Violin plot showing quantification of notochord lesions and rescue of notochord phenotype in *kita-RAS* (control for injections) (*n*=140) and *kita-RAS* injected with MO (*n*=41) or CRISPR (*n*=105) in comparison to *kita* (control) (*n*=52). Note that MO rescued the notochord phenotype, whereas CRISPR injections only partially rescued the notochord. In B, C, E and H, we used nonparametric, one-way ANOVA, Kruskal–Wallis test, followed by Dunn's multiple comparison test. *P*-values are shown when significant (*P*<0.05). In B, C and E, data are mean±s.d., generated in Prism 8. H was generated in Python.

To further test whether the increased innate inflammatory response triggers the proliferation of neoplastic cells leading to wounds in the notochord, we transiently delayed innate immune cell development by injecting *pu.1* (also known as *spi1b*) and *gcsfr* (also known as *csf3r*) morpholinos (MOs) (double knockdown) into the one-cell-stage embryos generated by incrosses of *kita-RAS-GFP* fish (Fig. S3). Combined *pu.1* and *gcsfr* MO injections are used to transiently arrest myeloid lineage development in larval zebrafish until at least 4 dpf, therefore generating larvae lacking neutrophils and macrophages (Feng et al., 2012; Liongue et al., 2009; Rhodes et al., 2005). We confirmed the efficiency of our MO experiment by injecting fish carrying labelled neutrophils and macrophages at 3 dpf [Tg(*lyz:DsRed*;*mpeg:FRET*)] (Fig. S3B). Blocking the development of inflammatory cells in *kita-RAS* resulted in a reduction of larvae exhibiting wounded (>5 lesions) notochordal phenotype from 44.37% (control MO) to 8.56% (*pu.1*+*gcsfr* MO) (*P*<0.0001) at 3 dpf (Fig. S3C,D). In addition, fish with affected notochord (8.56%) in the *pu.1*+*gcsfr* MO group showed a less severe (≤5 lesions) phenotype in comparison to the control MO group, suggesting that incomplete ablation of inflammatory cells can ameliorate chordoma. To complement our MO experiment, we used the CRISPR/Cas9 system to target *pu.1* and *gcsfr* simultaneously. We were able to cause mutations with an efficiency rate of 80%, validated by fragment length analysis, for each individual gene, at 5 dpf. We analysed *kita-RAS* larvae from MO and CRISPR injections side by side at 5 dpf (Fig. 4A). CRISPR injections led to a significant reduction in numbers of neutrophils (*P*=0.0012) and macrophages (*P*=0.0478), but this reduction was not as pronounced as that observed from MO injections (*P*<0.0001) (Fig. 4B-D). MOs also led to a significant reduction in the proliferation of notochord and notochord epithelium cells in *kita-RAS* (Fig. 4E,F). Although CRISPR injections reduced cell proliferation, the resulting cell proliferation was not statistically different from that of *kita-RAS* (*P*=0.2422) (Fig. 4E,F). In comparison to non-affected notochords from controls, fluorescent stereomicroscopy pictures from *kita-RAS* wounded notochords displayed different profiles of average pixel intensity. Notochordal lesions are detected by increased pixel intensity and enlargement of peak areas (Fig. 4G). This unbiased method allowed us to quantify the severity of notochordal wounds among the studied groups and to analyse whether we could rescue the affected notochordal phenotype upon MO and CRISPR injections. We compared *kita* (control), *kita-RAS* and *kita-RAS* injected with either MO or CRISPR. Similar to our cell proliferation experiment, we detected a partial notochordal rescue with CRISPR injections and significant rescue with MO injections (Fig. 4H). Therefore, we have shown that the increase in neutrophils and macrophages contributes to proliferation of cancer cells in the notochord, and modulation of inflammatory cells could prevent clonal expansion and chordoma development, similar to what has been previously shown for melanomas (Feng et al., 2012).

### Abnormal pattern of vertebral segmentation and mineralisation in *kita-RAS* fish

**Fig. 5.**
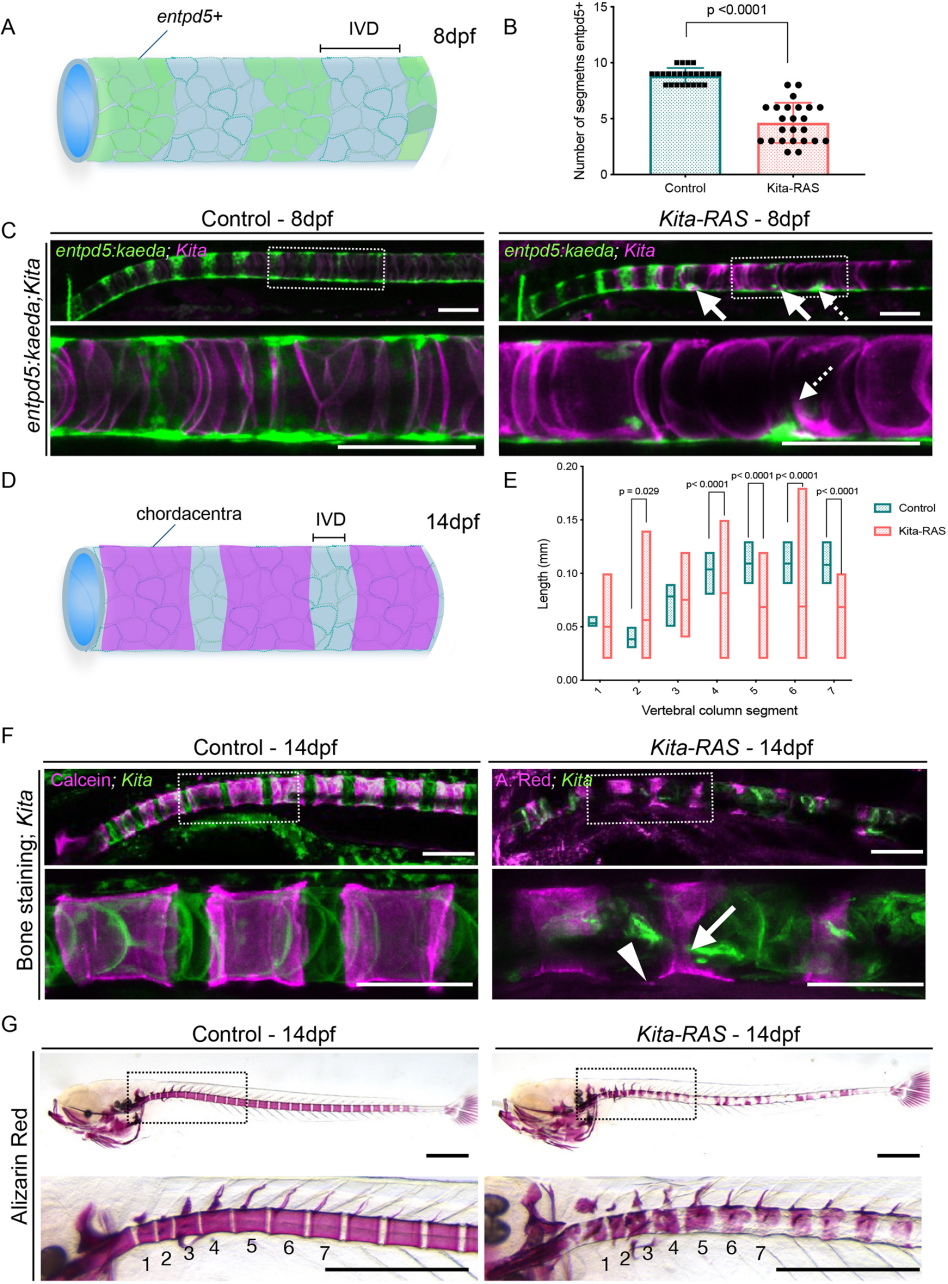
**Notochord and sheath destabilisation interfere with vertebral column segmentation and mineralisation in *kita-RAS*.** (A) Diagram illustrating the expression of *entpd5(+)* in controls. These domains are interspaced by *entpd5(−)*, which will form the intervertebral discs (IVDs), under normal situation. (B) Numbers of *entpd5^+^* segments counted from zebrafish at 5 dpf with length between 3.8 mm and 4.1 mm. Note the slow formation of segments in *kita-RAS* (*n*=24) in comparison to controls (*n*=25). Unpaired, nonparametric *t*-test and Mann–Whitney test were used. Data are mean±s.d. (C) *entpd5* expression in control (*kita:mCherry*) and *kita-RAS* at 8 dpf. Maximum projections from *z*-stacks of notochord (*kita*) and *entpd5:kaeda* are shown for merged channels. Selected regions (dashed line boxes) are shown at higher magnification. Note abnormal expression pattern of *entpd5* (arrows) coinciding with the wounded region (dashed line arrows). (D) Diagram illustrating where the notochord sheath will mineralise from *entpd5^+^* regions and form the chordacentra (vertebral primordium). (E) The lengths of the first seven segments of the vertebral column were measured from controls (*n*=24 fish) and *kita-RAS* (*n*=23 fish) of similar total length (5 mm≤fish length<6 mm) at 14 dpf. Graph displays seven segments and their lengths. Note the high variability in *kita-RAS.* Unpaired, nonparametric, multiple *t*-tests were performed for statistical analysis. Lines indicate the means. *P*-values are shown when significant (*P*<0.05). (F) Alizarin Red S and Calcein Green (bone staining) were used to visualise the mineralised chordacentra at 14 dpf in controls and *kita-RAS*. Maximum projections from confocal images are shown for merged channels. Selected regions (dashed line boxes) are shown at higher magnification. Incomplete mineralisation of the chordacenta (arrow) and ectopic mineralisation towards the IVD domain (arrowhead) were detected in *kita-RAS*. Scale bars: 100 µm. (G) Alizarin Red S staining was performed on 14 dpf fixed samples for measurements of segment lengths. Note uneven mineralisation of the segments. Selected regions (dashed line boxes) are shown at higher magnification. The first seven vertebral segments are indicated. Scale bars: 500 µm.

It has been demonstrated that notochord damage can lead to defective patterning of the vertebral column (Lopez-Baez et al., 2018; Fleming et al., 2004; Nguyen-Chi et al., 2014). Given that *kita-RAS* causes cellular changes and a wound-like response in the notochord, we questioned whether these events might have a downstream impact on vertebral column segmentation. We crossed *kita-RAS* to Tg(*entpd5:kaeda*), an early marker of notochord segmentation and biomineralising activity. Entpd5 (also known as Entpd5a) hydrolyses nucleoside triphosphates, providing local inorganic monophosphate for biomineralisation (Dallas and Bonewald, 2010; Huitema et al., 2012). During development of the vertebral column, *entpd5* is expressed in alternating segments of the sheath, which will form the mineralised chordacentra; the interdomains will develop into IVDs (Fig. 5A,D) (Wopat et al., 2018). We analysed larvae at 8 dpf, at a stage when segmentation has started but is not yet finalised. A delay in chordacentra formation was observed in *kita-RAS*, compared to control of similar range in length (3.8-4.1 mm) (Fig. 5B). Regions in which the notochord cells were compromised in *kita-RAS* coincided with mispatterning and ectopic expression of *entpd5:kaeda* (Fig. 5C). Expansion of the domain of each segment was observed ectopically in the future IVD area. These results indicate that cellular abnormalities of the notochord sheath compromise the differentiation of *col9a2^+^* sheath cells towards expression of *entpd5* in pre-determined chordacentra domains during segmentation. Moreover, our findings suggest a role of the sheath layer and notochordal cells in domain specification. A major advantage of our *kita-RAS* model in comparison to other notochord induced RAS models (Burger et al., 2014; D'Agati et al., 2019; Distel et al., 2009) is the fish survival to adult stages, beyond the stages of development that have been previously reported. This allowed us to study the effect of pre-neoplastic cells on skeletal formation and homeostasis. To check for abnormalities in mineralised vertebral column segments, we used *in vivo* and *ex vivo* Alizarin Red S staining in 14 dpf fish. We detected abnormal and uneven mineralisation of the chordacentra along the whole notochord, compromising the length and shape of the segments and future IVD domains (Fig. 5E-G). We measured the lengths of the first seven mineralised vertebral segments from fish displaying similar sizes (5 mm≤fish length<6 mm) (Fig. 5G). *kita-RAS* showed high variability and overall reduced length of segments (Fig. 5E). Our results indicate that the presence of notochord cancer cells leads to a wounded notochord sheath that modifies vertebral column segmentation pattern through ectopic activation of *entpd5* and subsequent mineralisation, which ultimately may cause vertebral fusions.

### Transformed notochord cells lead to vertebral column fusions and clefts

**Fig. 6.**
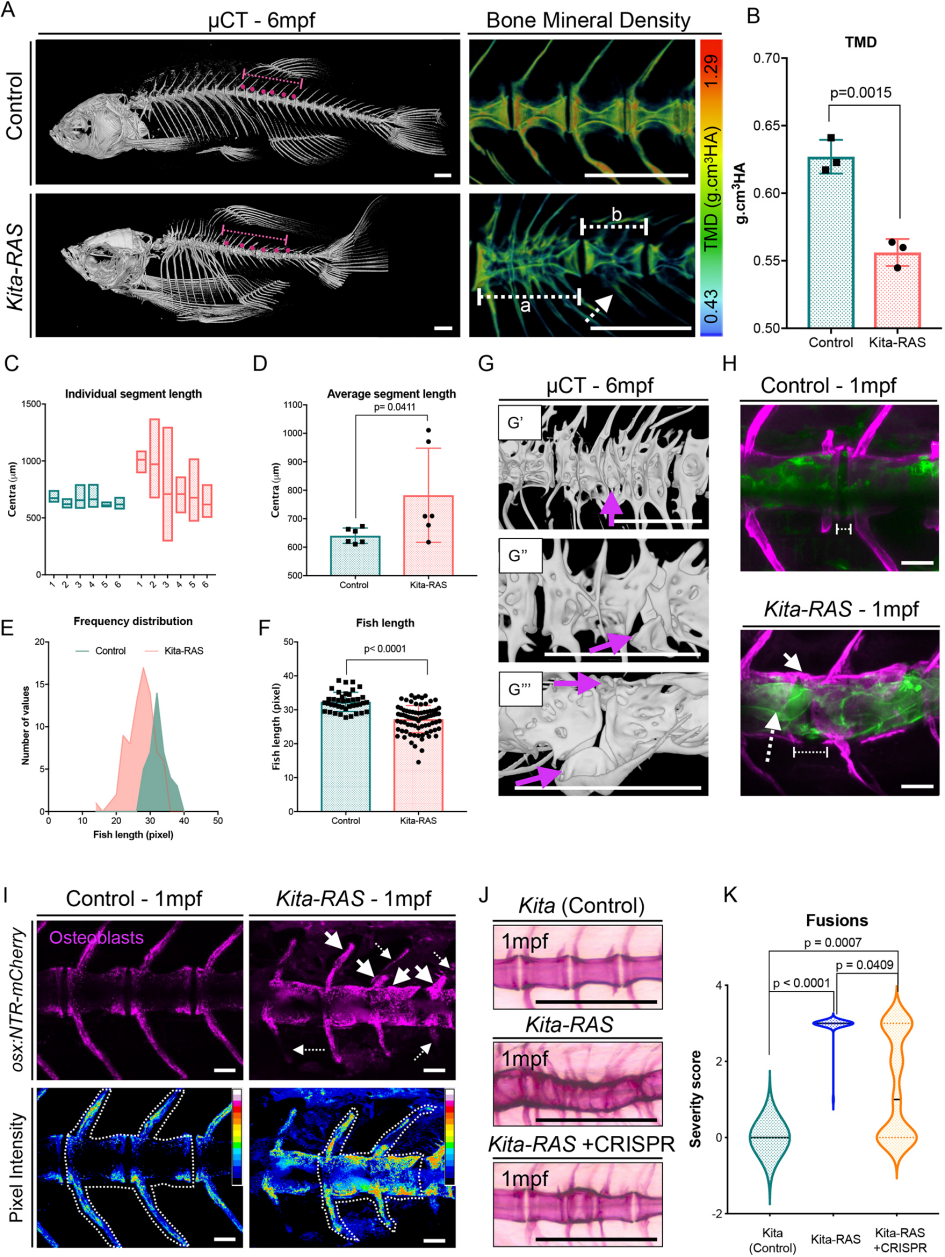
**Transformed notochord cells lead to fusions and vertebral clefts,** **which can be rescued by immune cell modulation.** (A) µCT images of adult (6-month-old; 6 mpf) control (*kita-mCherry*) and *kita-RAS*. Note severe fusions and shortening of the fish length in *kita-RAS*. A zoomed region, colour coded for bone mineral density [tissue mineral density (TMD); in g/cm^3^ hydroxyapatite (HA)], is shown as an example. Note the decreased mineral density in *kita-RAS*. Fusions compromising two (white dashed line, b) to several vertebrae (white dashed line, a) are shown. The arches are also compromised (white dashed line arrow). Scale bars: 500 µm. (B) TMD calculation. Unpaired two-tailed Student's *t*-test was used as a statistical test (two vertebrae per fish were analysed; control *n*=3 fish, *kita-RAS n*=3 fish). (C) Frequency distribution of the length of six consecutive segments, separated by a defined IVD space, were measured in Amira using 3D perspective measurement. The studied region is shown with a dashed line and magenta dots in A. *kita-RAS* show high variability in length of segments. (D) The average segment length was increased in *kita-RAS*. Six vertebrae per fish were analysed; control *n*=3 fish, *kita-RAS n*=3 fish. Unpaired, nonparametric *t*-test (Mann–Whitney test). (E) Frequency distribution of fish length in controls and *kita-RAS* measured in pixels, from X-ray images. (F) Fish lengths (measured in pixels) of controls (*n*=40) and *kita-RAS* (*n*=78). Unpaired, nonparametric *t*-test (Mann–Whitney test). (G) Higher-resolution µCT images to show abnormalities in detail. G′, fusions of several vertebrae and hemicentra (arrow). G″, lateral view of a hemicentra (arrow). G‴, ventral view of a hemicentra (arrows). Scale bars: 500 µm. (H) One-month-old (1 mpf) control (*kita-mCherry*) and *kita:RAS-GFP* stained with Calcein Green and Alizarin Red S, respectively, to label the bone (magenta). In *kita-RAS*, a hyperplastic notochord cell is indicated with a white dashed line arrow, mineralised IVD is indicated with a white arrow, and a region of incomplete mineralisation and future cleft is marked with a dashed line. Note that notochordal cells fail to organise in IVD domains. Scale bars: 50 µm. (I) 1 mpf control and *kita:RAS-G*FP showing osteoblasts [Tg(*osx:NTR-mCherry*)]. Arrows indicate regions of increased osteoblasts; dashed line arrows show regions lacking osteoblasts and abnormal growth of arches. Pictures were processed to show pixel intensity (blue=low intensity), to visualise where osteoblasts are highly expressed. Two vertebrae in each fish were selected for quantification of mean pixel intensity. (J) Alizarin Red S staining of 1 mpf *kita*, *kita-RAS* and *kita-RAS+CRISPR.* Note an intermediate (less severe) phenotype in *kita-RAS+CRISPR*, suggesting rescue of bone phenotype. (K) Violin plot to show the distribution of vertebral column severity scores, from 0 (less severe) to 3 (most severe), in *kita* (*n*=47), *kita-RAS* (*n*=44) and *kita-RAS+CRISPR* (*n*=83). One-way ANOVA and Tukey's multiple comparisons test were used; *P*-values are indicated when significant. Scale bars: 50 µm.

Next, we sought to investigate the impact of pre-neoplastic cells in the vertebral column architecture. For that, we analysed the adult vertebral column, looking for resulting bone abnormalities. We used Alizarin Red S staining [controls *n*=10; *kita-RAS n*=10; 6 months post-fertilisation (mpf)], X-rays (controls *n*=40; *kita-RAS n*=78; 1-year-old fish) and micro-computerised tomography (μCT) (controls *n*=5; *kita-RAS n*=5; 6 mpf) to compare *kita-RAS* with control fish of the same age. Vertebrae fusions were found in 100% of *kita-RAS* and in 0% of controls (controls *n*=40; *kita-RAS n*=78) (Fig. 6; Fig. S4). Fusions involved two or more vertebrae along the vertebral column, leading to shortening of the total fish length. The fish with most fusions had the most reduced lengths (Fig. 6A,E; Fig. S4). The ribs were the most severely affected region of the vertebral column. We calculated the lengths of six consecutive mineralised segments of the vertebral column, separated by well-defined IVDs (Fig. 6A, dashed line region). Besides uncovering increased lengths of segments due to vertebral fusions (*P*=0.0411), it highlighted high variability within the same vertebral column region of *kita-RAS* fish, demonstrating that there was no common developmental pattern of fusions (Fig. 6C,D). Analysis of fish length from X-ray images reinforced the length reduction observed in *kita-RAS* (*P*<0.0001) (Fig. 6E,F). *kita-RAS* also displayed shape abnormalities of vertebrae and arches, including enlarged regions, broadening of arches and ectopic bone growth (Fig. S4). Enlarged areas were found in 40% of *kita-RAS* (Fig. S4B). Ectopic bone growth can be better visualised with higher resolution μCT (5 μm) (Fig. 6G) and Alizarin Red S staining (Fig. S4C). Clefts through the centra and hemicentrae were found in 70% of fish analysed. These resembled butterfly abnormalities as occasionally described in human vertebral columns (Katsuura and Kim, 2019), and malformations involving notochordal remnants (Fig. 6G‴) (Oner et al., 2006). When staining 1-month-old (1 mpf) *kita-RAS* with Alizarin Red S, we detected hyperplastic cells contributing to a chaotic notochord cell arrangement along the vertebral column and failure to organise in IVDs domains, revealing regions of incomplete mineralisation, originating clefts (Fig. 6H). To visualise osteoblasts, we crossed *kita-RAS* fish to Tg(*osx:NTR-mCherry*), an osteoblast reporter line, and analysed the vertebral column at 1 mpf. In controls, the osteoblasts were distributed evenly through the arches and centra; however, *kita-RAS* showed increased osteoblast signal and patchy distribution, with some regions displaying dense concentrations of osteoblasts and others lacking these cells. Quantification of osteoblasts was performed for two consecutive vertebrae in each fish (*n*=3), confirming an increase in osteoblasts in *kita-RAS* (*P*=0.0028) (Fig. S5D). Moreover, we detected irregular recruitment of osteoblasts to the chordacentra throughout the vertebral column. Thus, changes in the notochord led to abnormal osteoblast recruitment and behaviour. Next, we asked whether reduction of inflammatory cells could rescue the bone phenotype. We looked at the vertebral column of controls and *kita-RAS +* CRISPR (*pu.1*+*gcsfr*) fish at 1 mpf by Alizarin Red S staining. The severity of the vertebral column phenotype was scored depending on the number of fusions and clefts observed. *kita-RAS +* CRISPR (*pu.1*+*gcsfr*) partially rescue the vertebral column phenotype (Fig. 6J,K), with a subset of fish showing no fusions or clefts (Fig. 6K). Therefore, modulation of innate immune cells in our chordoma model prevents vertebral fusions and clefts.

### Compromised IVDs and impaired bone quality in adult *kita-RAS*

**Fig. 7.**
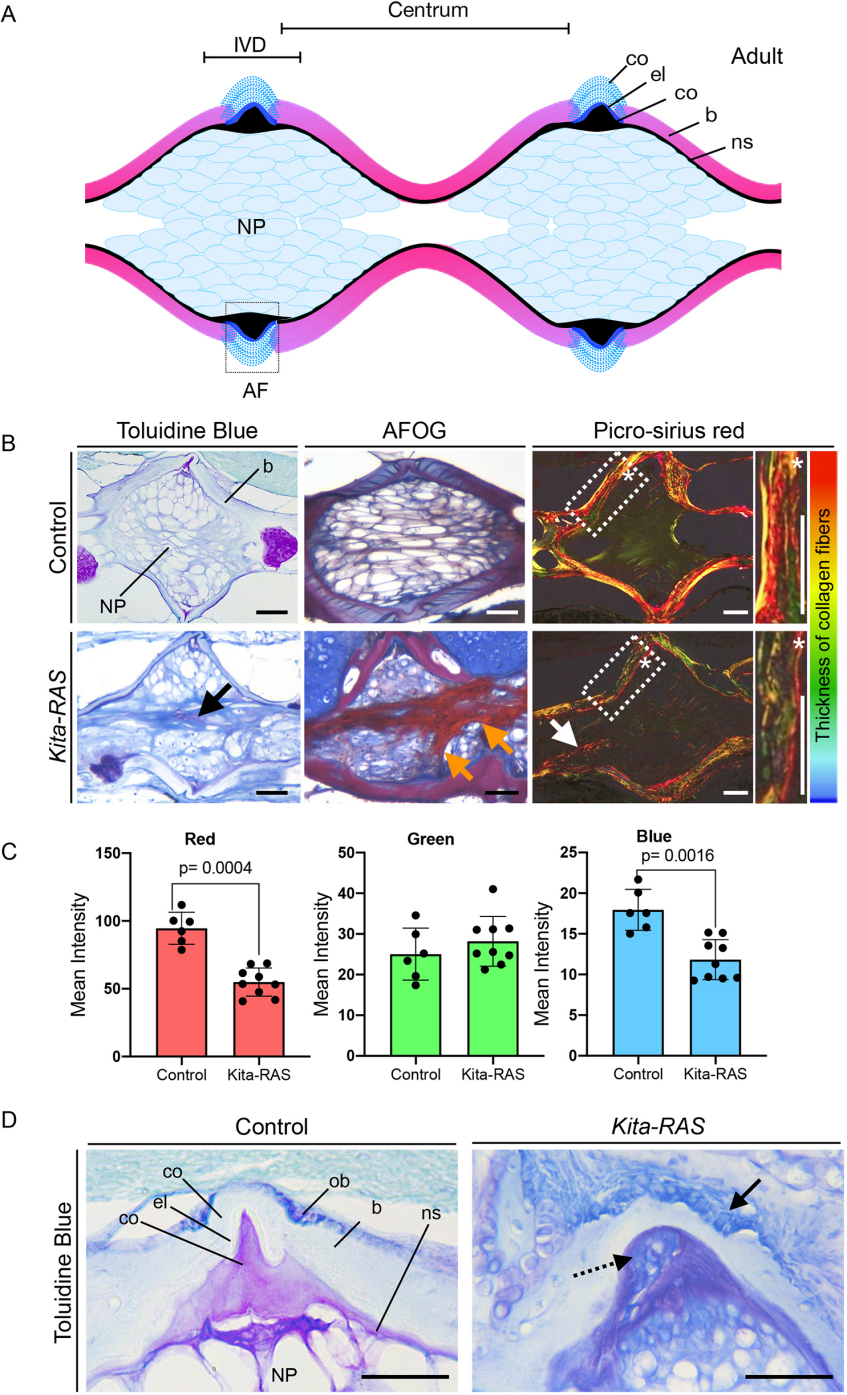
**Fibrotic nucleus pulposus and abnormal annulus fibrosus in *kita-RAS* resemble intervertebral disc degeneration.** (A) Schematic of a histological section of the vertebral column of zebrafish (off from the midline) showing two consecutive IVDs. AF, annulus fibrosus; b, bone; co, collagen layers; el, elastin layer; IVD, intervertebral disc; NP, nucleus pulposus; ns, notochord sheath. (B) Histological sections of adult control (*kita-mCherry*) and *kita-RAS* fish stained with Toluidine Blue (morphology), AFOG (fibrosis) and Picro-Sirius Red (fibrosis and collagen fibre thickness). Bone (b) and inner nucleus pulposus (NP) are indicated on the control Toluidine Blue picture. Abnormal fibrosis (black, orange and white arrows), cellularity and disorganisation of the NP were detected in *kita-RAS* fish. The regions within the dashed line boxes (Picro-Sirius Red staining) are shown at higher magnification to show the bone in detail. Asterisks were added to help with orientation, and they show the same position in lower- and higher-magnification pictures. Poor quality of bone can be measured by the tones of colours from Picro-Sirius Red staining. Thicker fibres are red and thinner fibres are blue/green (colour bar). (C) Collagen fibre quantification was performed by determining the means of pixel colours (red, green and blue) in the Picro-Sirius Red staining pictures. Note a reduction of thick (red) and very thin (blue) fibres in *kita-RAS* (*n*=9 vertebrae, *n*=3 fish) in comparison to controls (*n*=6 vertebrae, *n*=3 fish). Unpaired, nonparametric *t*-test and Mann–Whitney test were used. Data are mean±s.d.; *P*-values are indicated when significant (*P*<0.05). (D) Toluidine Blue staining to show details of the AF area in control (*kita-mCherry*) and *kita-RAS*. Note the loss of the layers of collagen and elastin in *kita-RAS* and disorganised and higher number of osteoblasts (arrow). Internal collagen layer is mixed with abnormal cells (dashed line arrow). Scale bars: 50 µm.

Embryonic notochordal cells contribute to the formation of the IVD NP, which plays an important role in regulating disc homeostasis (Choi et al., 2008). We sought to understand the impact of transformed notochord cells in the adult zebrafish IVD-equivalent regions and vertebral bone. By calculating bone mineral density, we detected a significant tissue mineral density (TMD) decrease in *kita-RAS* (*P*=0.0015) (Fig. 6A,B), indicative of impaired bone quality. We analysed histological sections of the adult vertebral column and observed highly fibrotic NP, similar to IVDD, with disorganised cellularity found in enlarged vertebrae (Fig. 7A,B). Fibrosis was detected in proximity to the notochord sheath layer. AFOG and Picro-Sirius Red staining confirmed fibrosis and connectivity with the notochord sheath, showing increased collagen content and increased collagen fibre thickness (Fig. 7B,C). In contrast to IVDD, dehydration was not observed in *kita-RAS* NP, as an increase in glycosaminoglycans was detected (Fig. S5). Additionally, despite fibrosis and disorganisation of the NP, owing to cell transformation, we did not observe IVD calcification, a feature commonly found during IVDD and ageing (Novais et al., 2020a). The outermost component of the discs, the AF, was replaced by bone in IVDs that were compromised by fusions. The structured layers of collagen and elastin that form the zebrafish AF were completely lost in some of the IVDs (Fig. 7D). Interestingly, disorganised and increased number of osteoblasts were detected in the IVD region, corroborating altered osteoblast activity at the endplates of adult fish. The balance between osteoblasts and osteoclasts is key in bone homeostasis and control of bone density. Moreover, osteoclasts are derived from the same cell lineage of macrophages. We performed whole-mount tartrate-resistant acid phosphatase (TRAP) staining to visualise osteoclast activity. Quantification of TRAP staining revealed exacerbated bone resorption in *kita-RAS* (*P*=0.0026), especially in affected areas of the vertebral column (Fig. S5B,C). Picro-Sirius Red staining suggested a reduction in collagen fibre thickness in the bone (centra). We quantified the mean intensity of red, green and blue pixels from pictures stained with Picro-Sirius Red. We detected a significant reduction in red (*P*=0.0004) and blue (*P*=0.0016) pixels, indicating abnormal fibre organisation and confirming bone quality impairment in *kita-RAS* (Fig. 7C). We conclude that transformed cells in the notochord lead to vertebral column and IVD abnormalities affecting the NP and AF, impairing osteoblasts and osteoclast activity, consequently altering bone homeostasis in zebrafish.

## DISCUSSION

“Tumours are wounds that do not heal” was postulated in a classic work published by Harold Dvorak in 1986 (Dvorak, 1986). Dvorak recognised that the composition of the tumour stroma strongly resembled healing skin wounds, suggesting activation of the wound-healing response in the host. Moreover, cancer is frequently the consequence of chronic inflammatory disease (Schafer and Werner, 2008). Given the confined nature of notochordal cells during development of the vertebral column, would pre-neoplastic notochordal cells trigger chronic inflammation as other cancers do? And what is the impact of transformed cells in disc and bone homeostasis? By demonstrating that transformed notochord cells provoke chronic notochordal wounds and activate wound response mechanisms in zebrafish, leading to inflammation, vertebral column abnormalities and impairment of disc and bone homeostasis, we demonstrated parallels between wound repair, cancer and IVDD in a zebrafish chordoma model.

The *UAS:EGFP-HRASV12* transgene has been successfully used to transform notochordal cells and melanoblasts, contributing to *in vivo* modelling of chordomas and melanomas (Burger et al., 2014; Feng et al., 2010; Santoriello et al., 2010; D'Agati et al., 2019). Here, we made use of the robustness of RAS expression systems to efficiently induce chordomas, using the stable line *kita-RAS*, an adult melanoma model with notochordal RAS expression. *kita-RAS* caused similar larval notochord morphopathological changes as previously described for *twhh:Gal;UAS:HRASV12* and *4465:Gal;UAS:HRASV12* (Burger et al., 2014), serving as tools to investigate neoplastic notochord cells in adults. Although *kita-RAS* has been extensively used to study melanomas, the vertebral column can still be studied in adult fish without complications of skin tumour, as only ∼20% of adult fish develop melanomas (Anelli et al., 2009). Despite being unlikely, the involvement of melanocytes in the advancement of chordoma cannot be ruled out from our model. Owing to melanoma active interaction with immune cells, exacerbated immune activity could possibly lead to worsening of the notochordal phenotype, and it should be further investigated. Alternatively, *kita-RAS* when crossed with a pigment-free line, such as *casper* (complete lack of melanophores and iridophores) or *nacre* (mutation in *mitfa*) (White et al., 2008) can prevent melanoma development. As for *UAS:EGFP-HRASV12* chordoma models, a limitation of the melanoma model is the fact that mutations of RAS members are not common in chordoma. However, RAS-transformed cells lead to activation of downstream signalling driven by EGFR, a cell surface receptor highly involved in chordomas, mimicking upstream receptor tyrosine kinase (RTK) activation (Burger et al., 2014). D'Agati et al. (2019) recently demonstrated that although *brachyury* (*tbxt*) overexpression did not have a tumour-initiating potential to transform notochord cells, when the authors tested RTK, including EGFR, they were able to trigger notochord hyperplasia, suggesting RTK signalling as a possible initiating event in chordoma.

**Fig. 8.**
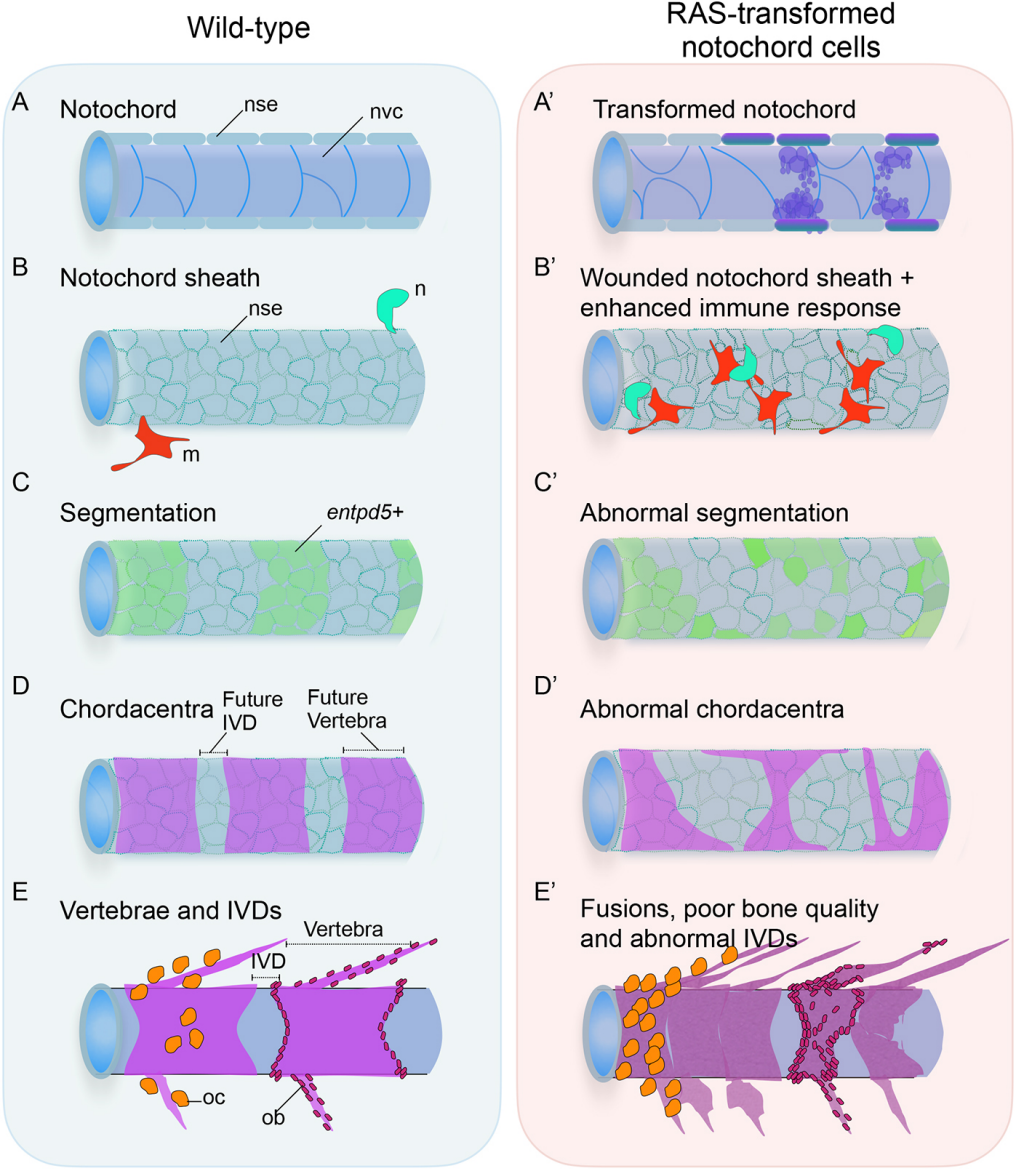
**Pre-neoplastic notochord cells drive abnormal vertebral column development and interfere with bone homeostasis in zebrafish.** (A) In wild-type zebrafish, the notochord is formed by a notochord sheath epithelium (nse) wrapping notochord vacuolated cells (nvc). (B) Innate immune cells, in particular neutrophils (n) and macrophages (m), are not directed to the notochord and they do not trespass the ns. (C) The segmentation of the notochord to form the future vertebrae and IVDs starts with differentiation of notochord sheath cells to express *entpd5* in interspaced domains. (D) These segments will mineralise (chordacentra) and originate individual vertebrae; intersegment regions will form the IVDs. (E) Osteoblasts (ob) and osteoclasts (oc) are evenly distributed in the centrae and arches. (A′) When RAS is expressed in the notochord cells, transformed vacuolated cells collapse and a fibrous ‘scar’ tissue is formed. (B′) The notochord sheath layer is destabilised, triggering a prolonged recruitment of neutrophils and macrophages. (C′) The notochord sheath cells fail to differentiate and to express *entpd5* in specific domains, showing a delay and abnormal pattern of expression. (D′) This leads to abnormal chordacentra formation, consequently leading to (E′) fusions, clefts and abnormalities in the adult vertebral column. IVDs are lost due to fusions. Osteoblasts and osteoclasts are distributed disorderly in centra and arches and in higher numbers. Moreover, pre-neoplastic cells continue to adulthood, leading to NP abnormalities and poor bone quality. Chordoma development and bone phenotype can be controlled by immunomodulation of neutrophils and macrophages.

Although human chordomas are thought to originate from hyperplasia of notochordal remnants, benign notochordal remnants are occasionally found and are associated with vertebral abnormalities, such as vertebral clefts and bifurcations (Oner et al., 2006). When we looked at the adult *kita-RAS*, we observed vertebral clefts and hemivertebrae that recapitulate human vertebral column abnormalities. However, vertebral malformations might not be a direct effect of pre-neoplastic notochordal cells, but a result of abnormal notochordal cell behaviour. Recent studies have shown that notochord vacuoles function as a hydrostatic scaffold that guides symmetrical growth of vertebrae and spine formation. Vacuole fragmentation caused by mutations in *dstyk* (*spzl* mutant) resulted in vertebral centra malformation and scoliosis (Bagwell et al., 2020; Sun et al., 2020). Similar to our observations, these studies evidenced that abnormal behaviour of notochord vacuolated cells is associated with vertebral malformations like those of notochordal remnants in human. Furthermore, hemivertebrae and clefts were systematically found in another mutant, *spondo*, carrying a mutation in *cmn* (encoding Calymmin, a teleost-specific extracellular matrix protein with weak similarity to Elastin, and expressed in the notochord sheath), owing to abnormalities in the notochord sheath layer (Peskin et al., 2020). Here, we demonstrated that destabilisation of the notochord vacuolated cells also triggered cellular changes in the notochord sheath layer (Fig. 8), revealing double and overlapping routes in which notochord neoplastic cells compromise the formation of the vertebral column: the inner vacuolated cells and the outer notochord sheath cells.

Notochord damage also leads to vertebral column abnormalities, including fusions and segmentation mispatterning (Lleras Forero et al., 2018; Wopat et al., 2018; Pogoda et al., 2018). We showed that *kita-RAS* mimicked notochordal damage and induced repair mechanisms, as demonstrated by activation and invagination of *col9a2^+^* notochord sheath cells and expression of *wt1b* in wounded areas, as previously described for notochordal wounds (Lopez-Baez et al., 2018; Garcia et al., 2017). Our findings suggest a key role of the notochord sheath and wound repair in chordoma. Interestingly, when RAS is activated in the notochord sheath specifically with *col2a1a* driving RAS, it also causes chordomas (D'Agati et al., 2019), sustaining a key role of the sheath layer in zebrafish chordomas. As neoplastic cells are continuously modifying the notochord, this causes wounds that seem to progress and remain chronic or unresolved. We showed for the first time that wounding provoked by transformed notochord cells triggers the recruitment of neutrophils and macrophages. Innate immune cells not only were present in higher number but changed their behaviour by prolonging their interaction time with the notochord sheath in wounded regions; in some cases they were able to breach the sealing membrane and achieve direct contact with cancer cells. It has recently been described that inflammatory cells make use of pre-existing holes in the basement membrane to gain access and reach pre-neoplastic cells in a melanoma model (van den Berg et al., 2019). In our chordoma model, inflammatory cells were observed in direct contact with pre-neoplastic cells in regions of severe notochord sheath wounds, which, similarly, may serve as breaches in the notochord sheath to allow neutrophils and macrophages to reach pre-neoplastic cells. The interactions between neutrophils/macrophages and transformed cells have been elegantly described for melanoma in zebrafish, with formation of cytoplasmic tether linking the two cell types and engulfment of transformed cells by neutrophils and macrophages (Feng et al., 2010). H_2_O_2_, a key damage signal directing recruitment of neutrophils to a wound, was also identified as the major component drawing recruitment of leukocytes to the transformed cells (Feng et al., 2010). Remarkably, when we depleted innate immune cells using MOs or CRISPR, we could rescue the notochord phenotype by inhibiting the aberrant proliferation of transformed cells, as demonstrated for melanoma (Feng et al., 2010), and partially rescuing the skeletal phenotype using CRISPR, by showing reduction of vertebral fusions, thus highlighting parallels between cancer and wound, and suggesting that immunomodulation might be a promising treatment for chordomas. Nguyen-Chi et al. (2014) showed that when zebrafish notochord is infected with *Escherichia* *coli*, strong and persistent recruitment of neutrophils and macrophages occurs. The authors also showed that *il1b* is partially required for recruitment of neutrophils but not macrophages. Fascinatingly, degranulation of neutrophils led to destruction of the host tissues and adult vertebral column defects, involving clefts and fusions. *il1b* morphants reduced neutrophil recruitment and prevented anterior notochord lesions. Altogether, inflammation appears to play an important role in controlling notochord damage and adult bone phenotype (Nguyen-Chi et al., 2014). By showing that mosaic ablation of innate immune cells by CRISPR ameliorate chordoma and the vertebral column phenotype, we highlighted potential opportunities for early intervention in the treatment of chordomas and vertebral column fusions.

*kita-RAS* fish displayed adult IVD abnormalities that resembled ageing zebrafish IVDD (E.K., unpublished data) with fibrotic NP and disorganised AF. Without parallel in zebrafish, we demonstrated that abnormalities in the early notochord cells and NP prime IVDD. Adult discs showed compromised notochord sheath, visualised by increased thickening of collagen fibres and fibre invasion towards the NP, indicating a likely involvement of wound repair mechanisms in adult discs and IVDD. Indeed, human orthologues encoding collagen type IX and collagen type XI are expressed in the notochord sheath and have been associated with IVDD in populational studies (Feng et al., 2016), which supports the involvement of the notochord sheath in IVDD in zebrafish. The inflammatory processes exacerbated by cytokines TNF-α and IL-1β are key events in IVDD (Risbud and Shapiro, 2014), they contribute to IVDD through degradation of extracellular matrix, and they are implicated in wounds and cancer. NP fibrosis during degeneration mimics wounds and fibrosis in other tissues (Novais et al., 2020a). *kita-RAS* also developed bone-quality impairment, emphasising NP modifications in regulation of bone homeostasis, suggesting changes in bone metabolic markers during chordomas. We detected increased osteoclast activity and chaotic osteoblasts at the endplates, in addition to osteoblast behaviour abnormalities and abnormal bone homeostasis. Osteoclasts share a common cell lineage with macrophages, and transdifferentiation of macrophages to osteoclasts has been reported (Pereira et al., 2018), suggesting opportunities to treat the bone phenotype through modulation of inflammation. In conclusion, using zebrafish, we showed parallels between chordomas, IVDD and wound repair, highlighting inflammation as a common event for potential therapeutic intervention.

## MATERIALS AND METHODS

### Zebrafish husbandry and lines

Zebrafish were housed as described (Westerfield, 2000). Transgenic lines included Tg(*kita:Gal4;UAS:mCherry;UAS:HRASG12V-GFP*) (Feng et al., 2010; Santoriello et al., 2010) and Tg(*kita:Gal4;UAS:mCherry;UAS:mCherry-HRASG12V*) (van den Berg et al., 2019), which were incrossed to obtain Tg(*kita:Gal4;UAS:HRASG12V-GFP*) and Tg(*kita:Gal4;UAS:mCherry-HRASG12V*), referred to as *kita-RAS*, and Tg(*kita:Gal4;UAS:mCherry*) as controls; Tg(*lyz:DsRed*) (Hall et al., 2007); Tg(*mpeg:FRET*) (a gift from Stephen Renshaw at the University of Sheffield); Tg(*col9a2:GFPCaaX*) (Garcia et al., 2017); Tg(*wt1b:GFP*) (Perner et al., 2007); Tg (*entpd5:kaeda*) (Huitema et al., 2012); and Tg (*osx:NTR-mCherry*) (Singh et al., 2012). Animal experiments were ethically approved by the University of Bristol Animal Welfare and Ethical Review Body (AWERB) and conducted under a UK Home Office project licence.

### Cellular proliferation assay

Cellular proliferation was quantified using the Click-iT Plus EdU Alexa Fluor 647 Imaging Kit (Life Technologies, C10640). Larvae were immersed in Danieau's solution containing 100 μM EdU solution and incubated for 24 h or 48 h at 28.5°C before termination of the experiment at 5 dpf. Larvae were then fixed in 4% paraformaldehyde (PFA) for 2 h at room temperature with gentle shaking, washed with PBS solution containing 0.5% Triton X-100 (PBST) and 3% (w/v) bovine serum albumin (BSA), and permeabilised in PBST solution containing 1% dimethyl sulfoxide for 1 h at room temperature. For EdU detection, larvae were washed in PBST 3% BSA and incubated with the Click-iT Plus reaction cocktail containing Alexa Fluor 647 azide for 30 min at room temperature, in accordance with the manufacturer’s protocol. For quantification, EdU^+^ cells within and in proximity to the notochord were counted manually through the *z*-stacks from confocal images and similar areas of interest.

### Confocal imaging

Live zebrafish were mounted ventrally on coverslips in 1% low-melting-point agarose containing MS222 (for live samples) and imaged using a Leica TCS SP8 AOBS confocal laser scanning microscope attached to a Leica DMi8 inverted epifluorescence microscope using a 10× dry lens or 20× glycerol lens. The temperature in the chamber covering the microscope was maintained at 28°C. Movies were recorded at an interval time of 5.45 min or 3.75 min per frame and a total time of 60 min or 120 min for neutrophils and macrophages, respectively.

### Confocal post-image analysis

Image processing was performed using Fiji (Schneider et al., 2012). For the analysis of number and time of neutrophil/macrophage interactions with notochord sheath, neutrophils and macrophages were considered to be interacting with the notochord sheath when they were in direct surface contact with the sheath layer. The number of these interactions and their duration were manually quantified from time-lapse movies in a pre-defined region of the flank above the caudal hematopoietic tissue in the zebrafish larva, from the total field of view. Neutrophils, macrophages and notochord were identified by visualisation of their fluorescence in the fluorescent channel, and the notochord sheath was more accurately distinguished by visualisation in the brightfield channel. Movies were exported from Fiji as QuickTime movies to play at 3 frames/s. For analysis of osteoblasts, images were converted to 32-bit, applied LUT (16 colours), flattened and then saved as tiff images. The tiff files were imported to Fiji, and two consecutive vertebrae were selected using the freehand selection tool, from which the mean pixel intensity values were calculated. For analysis of the area of notochord sheath cells (*col9a2^+^*), *kita-RAS* notochord was divided into wound-proximal and wound-distal regions. Using the freehand selection tool in Fiji, the areas of ten cells were analysed per region, using ten fish for controls and *kita-RAS*.

### MO injections

Previously described MOs including *pu.1* MO (5′-GATATACTGATACTCCATTGGTGGT-3′) (0.2 mM) (Rhodes et al., 2005), *gcsfr* MO (5′-GAAGCACAAGCGAGACGGATGCCAT-3′) (0.3 mM) (Liongue et al., 2009) and a scrambled MO (5′-CCTCTTACCTCAGTTACAATTTATA-3′) (0.5 mM) (GeneTools, USA) were injected into one-cell-stage embryos, as previously described (Liongue et al., 2009; Rhodes et al., 2005; van den Berg et al., 2019).

### CRISPR/Cas9 injections

We used three synthetic guide RNAs (gRNAs) targeting each of the genes *pu.1* and *gcsfr*, ordered as crispr RNAs (crRNAs; Sigma-Aldrich). We used the same target sites for *gcsfr* as previously described (Yang et al., 2020), while for *pu.1* we targeted the same genomic region as previously described in a *pu.1* mutant (chr7:32655153-32655197) (Yang et al., 2020). Pu.1 target sequences were *pu.1* cr1 GAGGGATGTGATGGCTACCC, *pu.1* cr2 AGCTCTGTAAAGTGGCTCTC and *pu.1* cr3 GCCTGGGTCCATGAAATGGC. All six crRNAs (2 pg) were incubated with trans-activating crispr RNA (tracrRNA; 10 pg) and GeneArt Platinum Cas9 nuclease (Invitrogen) prior to injections. Injections were administered to one-cell-stage embryos as previously described (Brunt et al., 2017). To validate CRISPR efficiency, DNA was extracted from 12 individual injected larvae at 5 dpf, followed by PCR amplification with FAM-M13F primer and gene-specific primers, with each forward primer containing an M13 tail (*pu.1* F, 5′-TGTAAAACGACGGCCAGTCCGTGTCTAGATCACTCTTGGG-3′; *pu.1* R, 5′-AAACCAAACCATAAATGATTCGTTTT-3′; *gcsfr* F, 5′-TGTAAAACGACGGCCAGTGATTGCTGACGTAACTATTGTAC-3′; *gcsfr* R, 5′-CTCACATTTAAAGTCTTATCAG-3′). PCRs were submitted to fragment length analysis (ABI 3500) (Carrington et al., 2015). Controls were injected with Cas9 protein and SygRNA^®^ SpCas9 tracrRNA (10 pg) (Merck). Images of the notochord were acquired at 5 dpf using a Leica fluorescent stereomicroscope (MZ10F), followed by analysis of notochord lesions.

### Analysis of notochord lesions

Notochord images of 5 dpf larvae previously injected with MO or CRISPR were analysed using custom Python scripts and by implementing three steps. First, we detected pixels of the notochord through manually setting the value of the intensity threshold. Second, we fitted the pixels with a 6th order polynomial function to obtain the intensity profile along the notochord. Specifically, the intensity profile was measured along the polynomial fit inside the image, using the algorithm adapted from the scikit-image package (van der Walt et al., 2014), where we modified the function ‘profile line’ to work with a polynomial line. The average value of the intensity profile was used as a measurement of the severity of lesions within the notochord. Finally, the average intensity from the notochord was compared among the different groups. For statistical analysis, we used ANOVA and Kruskal–Wallis H-test, implemented in scipy (Virtanen et al., 2020). Dunn's method was used for multiple comparison test, implemented in scikit-posthocs (Terpilowski, 2019), *P*-values were adjusted with Bonferroni.

### Alizarin Red S and Calcein Green staining

Alizarin Red S staining was performed on fixed fish to label calcified tissues and carried out using standard protocols (Walker and Kimmel, 2007). Live Calcein Green or Alizarin Red S staining was carried out as previously described (Bensimon-Brito et al., 2016). Fish at 14 dpf were fixed in 4% PFA before Alizarin Red S staining. Pictures of the entire fish were taken under a Leica stereomicroscope. Total fish length and the length of the first seven vertebral segments were measured using Leica LAS X Software.

### Vertebral column severity scoring system

Alizarin Red S staining was performed on fixed samples of 1-month-old fish (1 mpf) (*kita* control *n*=47; *kita-RAS n*=61; *kita-RAS+CRISPR n*=97), and pictures were taken with a Leica stereomicroscope (MZ10F). The length of each fish was measured from the nose to the most posterior extremity of the vertebral column; the tail fin was not included in the measurement. Those fish in which the vertebral columns were not completely formed were excluded from our severity score analysis. The vertebral column severity scoring system was based on numbers of fusions and clefts identified in each fish. Fusions and clefts were scored independently. Score of 3, *n*≥5; score of 2, 3<*n*<5; score of 1, *n*≤3; score of 0, *n*=0 (*n*=number of fusions and clefts).

### Radiographs (X-ray)

Live 1-year-old fish were anaesthetised with MS222 and radiographed using a MultiFocus digital radiography system (Faxitron) under 2× zoom and using the following settings: 45 kV, 5 s exposure and 0.46 A. A total of 118 fish were X-rayed (controls, *n*=40; *kita-RAS*, *n*=78). Fish lengths were measured using Fiji (Schindelin et al., 2012) (in pixels), using images that were acquired under the same conditions.

### µCT

Six-month-old fish (6 mpf) were fixed in 4% PFA for 14 days, followed by sequential washes in ethanol and maintained in a 70% ethanol solution. µCT was performed using a Nikon X-TEK 225 HT CT scanner under an X-ray source of 130 kV, 53 µA without additional filters. Whole fish were scanned at a voxel size of 20 μm, and selected spine regions rescanned at 5 μm. Images were reconstructed using CT Pro 3D software (Nikon). Amira 6.0 (FEI) was used to generate 3D volume and surface renders for image acquisition. For calculations of TMD, defined as measurement restricted to within the volume of calcified bone tissue (Bouxsein et al., 2010), the centrae were segmented and the mean grey values retrieved. Grey values were calibrated with phantoms of known densities (0.25 g/cm^3^ and 0.75 g/cm^3^ HA) and used for density calculations, as previously described (Kague et al., 2019). Three fish from each group were used for TMD calculation.

### Histology

Adult fish (3 mpf; control, *n*=3; *kita-RAS*, *n*=3) were fixed in 4% PFA for 14 days, then decalcified in 1 M EDTA solution for 20 days at room temperature. Larvae (control, *n*=3; *kita-RAS*, *n*=4) were fixed for 2 h. Samples were dehydrated in ethanol and embedded in paraffin, and sagittal sections were taken at 8 µm thickness. Selected slides were de-waxed and stained with Toluidine Blue (Kague et al., 2018)**,** Alcian Blue, AFOG or Picro-Sirius Red, as performed elsewhere (Hayes et al., 2013). Images were acquired on a Leica DMI600 inverted microscope, using 20× and 40× oil objectives, LAS software and a DFC420C colour camera. Quantification of thickness of collagen fibre was done using Fiji (Schindelin et al., 2012), by selecting an area of interest within the bone, followed by measurement of mean intensity of red, blue and green pixels.

### TRAP staining

TRAP staining was performed in whole-mount 3-month-old fish (3 mpf) (control, *n*=4; *kita-RAS*, *n*=5) using an Acid Phosphatase, Leukocyte (TRAP) Kit (Merck, 387A), following the instructions provided by the manufacturer. Fish were fixed overnight in fixative solution (provided). Samples were washed for 15 min in distilled water, followed by permeabilisation using 1% trypsin in 30% borate solution at 37°C overnight. Fish were incubated in TRAP staining solution (provided) at 37°C for 6 h in the dark, followed by two washes of 10 min each in distilled water. Pigmentation was removed by incubating the specimens in 3% H_2_O_2_. Pictures were taken from dissected spines placed in 70% glycerol under a Leica stereomicroscope. Quantification of TRAP signal was performed using Fiji (Schindelin et al., 2012). Images were converted to 32-bit and LUT (physics) applied. We inverted the LUT, flattened the images and calculated the mean of red pixels, corresponding to high TRAP signal.

### Statistical analysis

GraphPad Prism 8 was used for statistical analyses. The statistical test used for each panel can be found in the corresponding figure legend. *P*<0.05 was considered statistically significant.

## Supplementary Material

Supplementary information
