# Supplementary material for: Transformed notochordal cells trigger chronic wounds in zebrafish, destabilizing the vertebral column and bone homeostasis
Source: Dis Model Mech. 2021 Mar 19;14(3):dmm047001. doi: 10.1242/dmm.047001 (PMC7988777; doi:10.1242/dmm.047001)
Supplement: Supplementary information [file dmm-14-047001-s1.pdf]

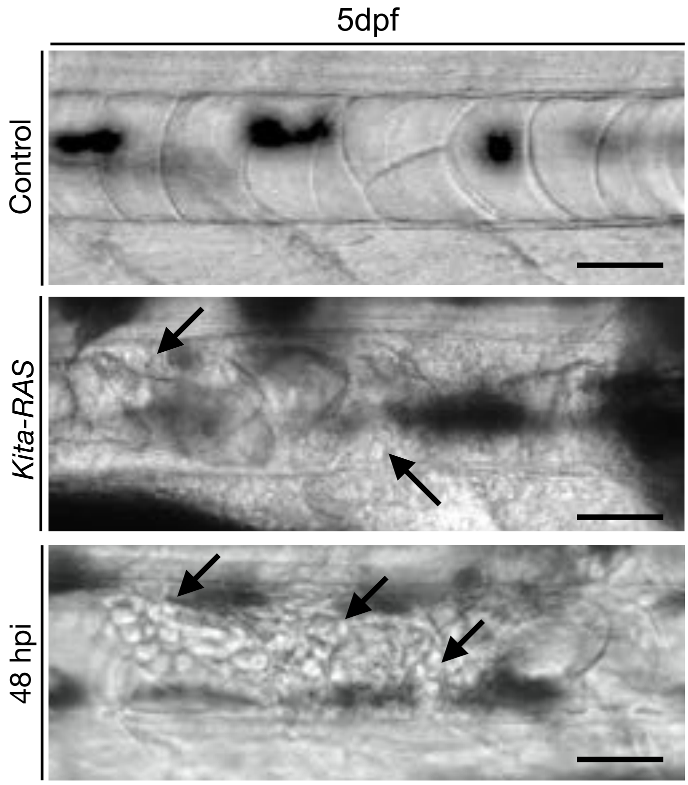

Figure S1. *Kita-RAS* induces notochord damages reminiscent of those from needle puncture wounded notochord. Transmitted light of 5dpf control (non-injured, *Kita-mCherry*), *Kita-RAS* and *Kita-mCherry* 48 hours post-injury (hpi). Note the increased amount of non-vacuolated cells (arrows) in the wounded region. Needle puncture was performed at 3dpf, as previously published (Lopez-Baez et al., 2018). Scale bars = 50 µm.

Disease Models & Mechanisms • Supplementary information

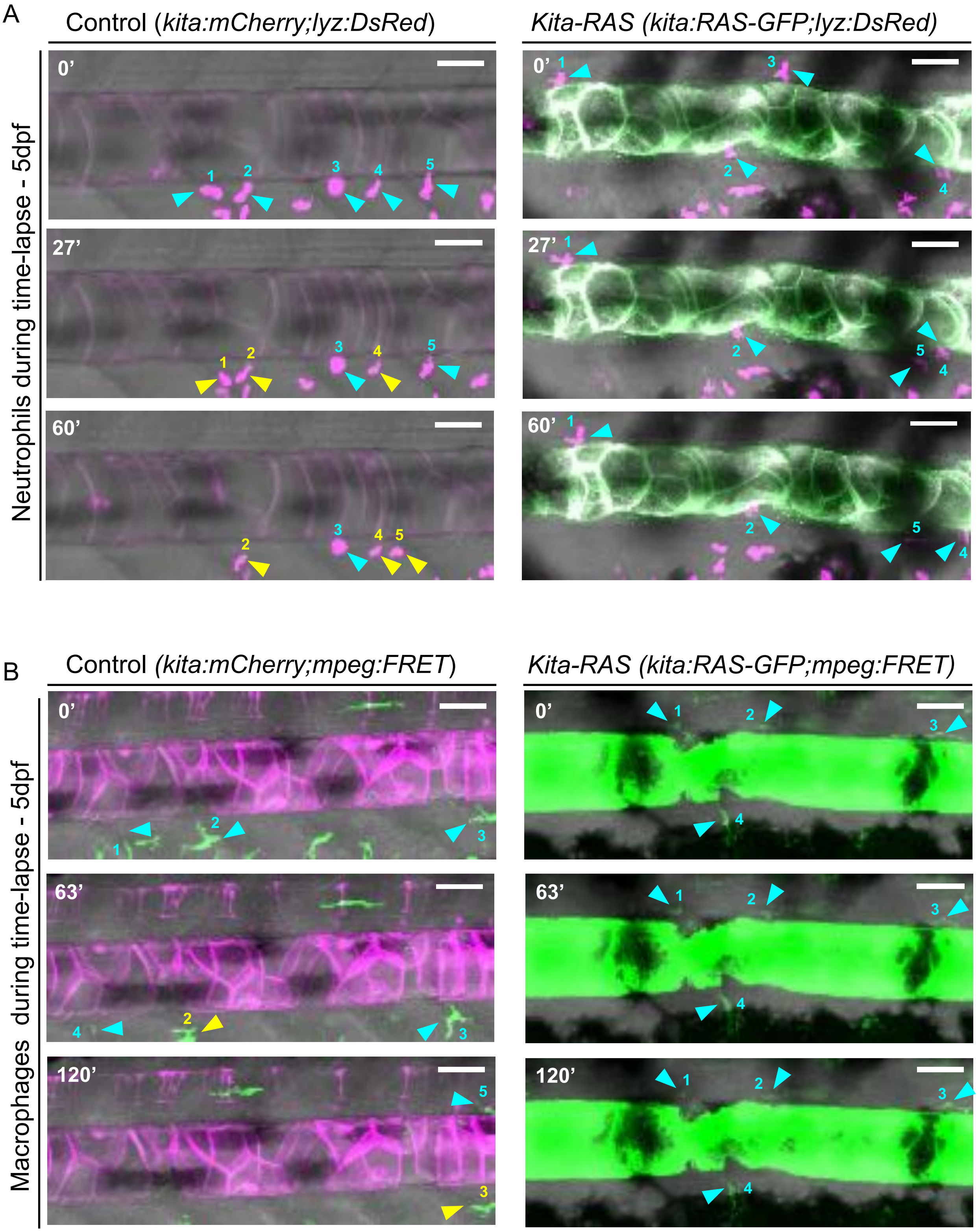

Disease Models & Mechanisms • Supplementary information

Figure S2. Increased number of neutrophils and macrophages interacting with the notochord sheath. A) Neutrophils in contact (cyan arrowhead) or not (yellow arrowhead) with the notochord sheath at each time point during the 60 min of time-lapse movies in control (*kita:mCherry;lyz:DsRed*) and Kita-RAS (*kita:RAS-GFP;lyz:DsRed)*. B) Macrophages in contact (cyan arrowhead) or not (yellow arrowhead) with the notochord sheath at each time point during the 120 min of time-lapse movies in control (*kita:mCherry;mpeg:FRET*) and *Kita-RAS* (*kita:RAS-GFP;mpeg:FRET*). Scale bars = 50 µm.

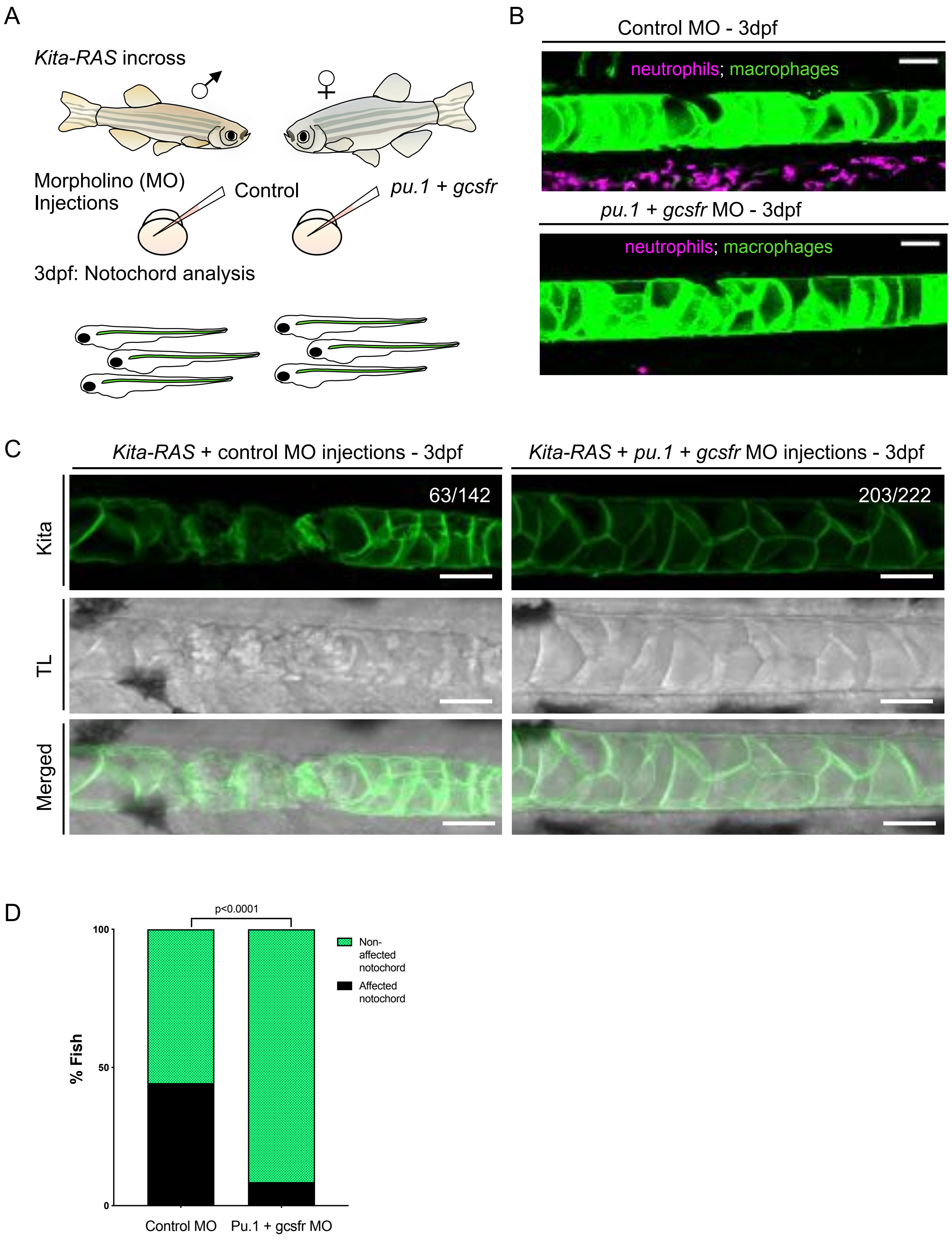

Disease Models & Mechanisms • Supplementary information

Figure S3. Modulation of neutrophils and macrophages by morpholinos rescues the normal notochord phenotype at 3dpf.

A) Schematic of rescue experiment. *Kita-RAS* was incrossed, embryos from the same cross were divided in two groups and each group injected with either control morpholino (scrambled sequence) or *pu.1* + *gcsfr* morpholinos (for depletion of neutrophils and macrophages) at 1 cell stage. The notochord was phenotypically evaluated at 3dpf. B) *pu.1+gcfsr* morpholinos effectively ablated macrophages and neutrophils in *Kita-RAS* (*kita:HRASG12V-GFP;lyz:DsRed;mpeg:FRET)* as shown by confocal images at 3dpf. Scale bars = 50 µm. C) Confocal images showing affected and non-affected notochord in control (63 out of 142) and *pu.1* + *gcsfr* (203 out of 222) groups, respectively. Images are shown with the GFP channel (RAS-GFP), transmitted light (TL) and merged. The numbers in the top right top corner indicate the number of fish with the phenotype as in the picture and the total number of fish injected. D) Percentage of fish at 3dpf that showed lesioned (> 5 lesions) (black) and non-lesioned (zero lesions) (green) notochord in the two groups: control (n = 142) and *pu.1* + *gcsfr* (n = 222). Note a significant increase in % of non-affected (normal notochord) fish in the *pu.1+gcsfr* group was observed. Fisher's exact test was used as statistical test. Scale bars = 50 µm.

Disease Models & Mechanisms • Supplementary information

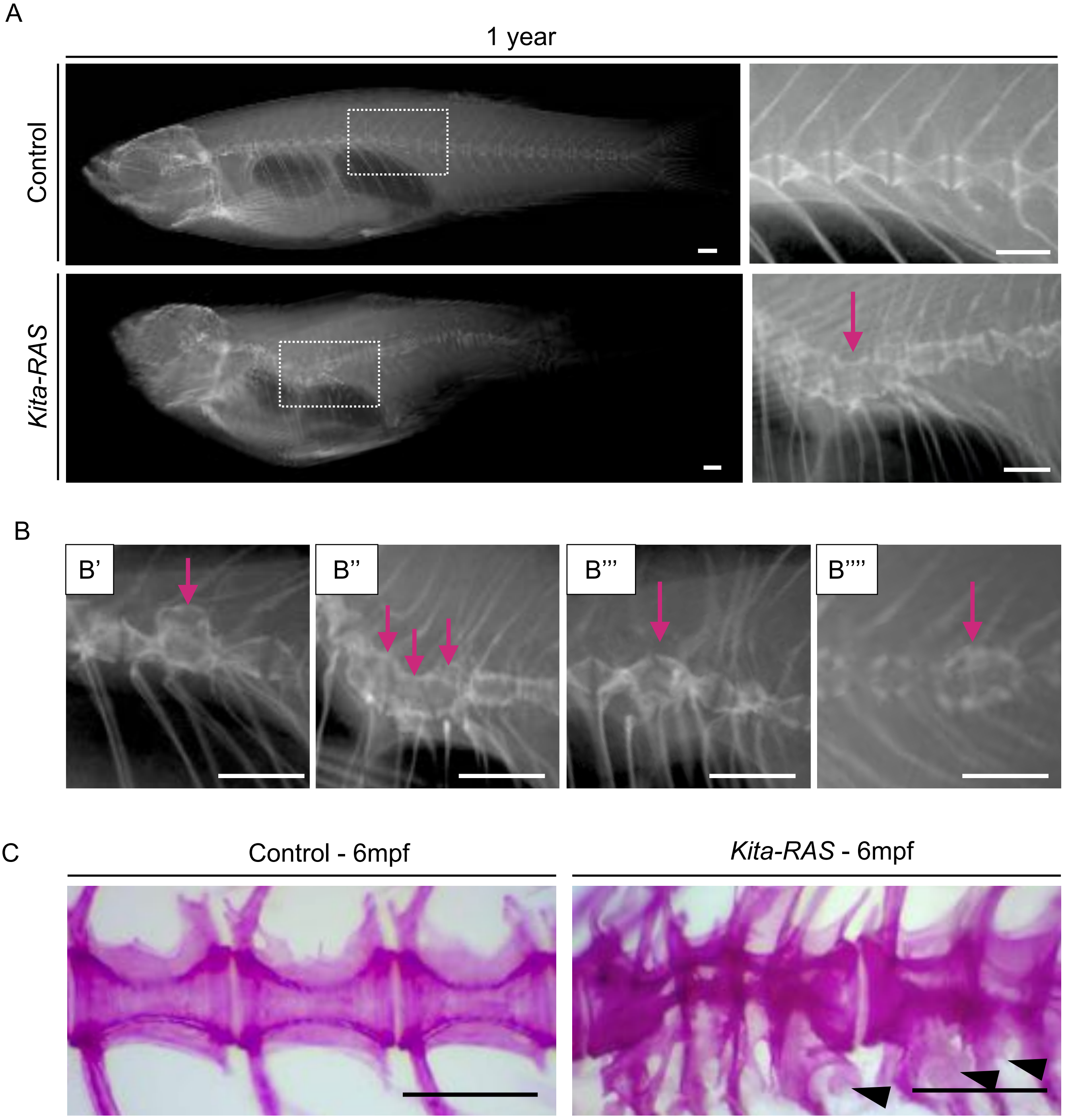

Disease Models & Mechanisms • Supplementary information

Figure S4. Transformed notochordal cells cause vertebral column abnormalities in adult fish. X-rays to compare *Kita-RAS* and control fish. Note the shortening of the body, fusions and abnormal arches. Selected region (box) was zoomed in for details. Enlarged vertebrae were observed (arrow). B) X-rays of *Kita-RAS* exemplifying enlarged vertebral column regions (arrows) detected in different *Kita-RAS* fish. Note evident enlarged vertebral regions in B’’ and B’’’’. C) Alizarin Red S staining to show details of trabeculation and abnormal bone growth in the arches of *Kita-RAS* (arrowheads). Scale bars = 500 µm.

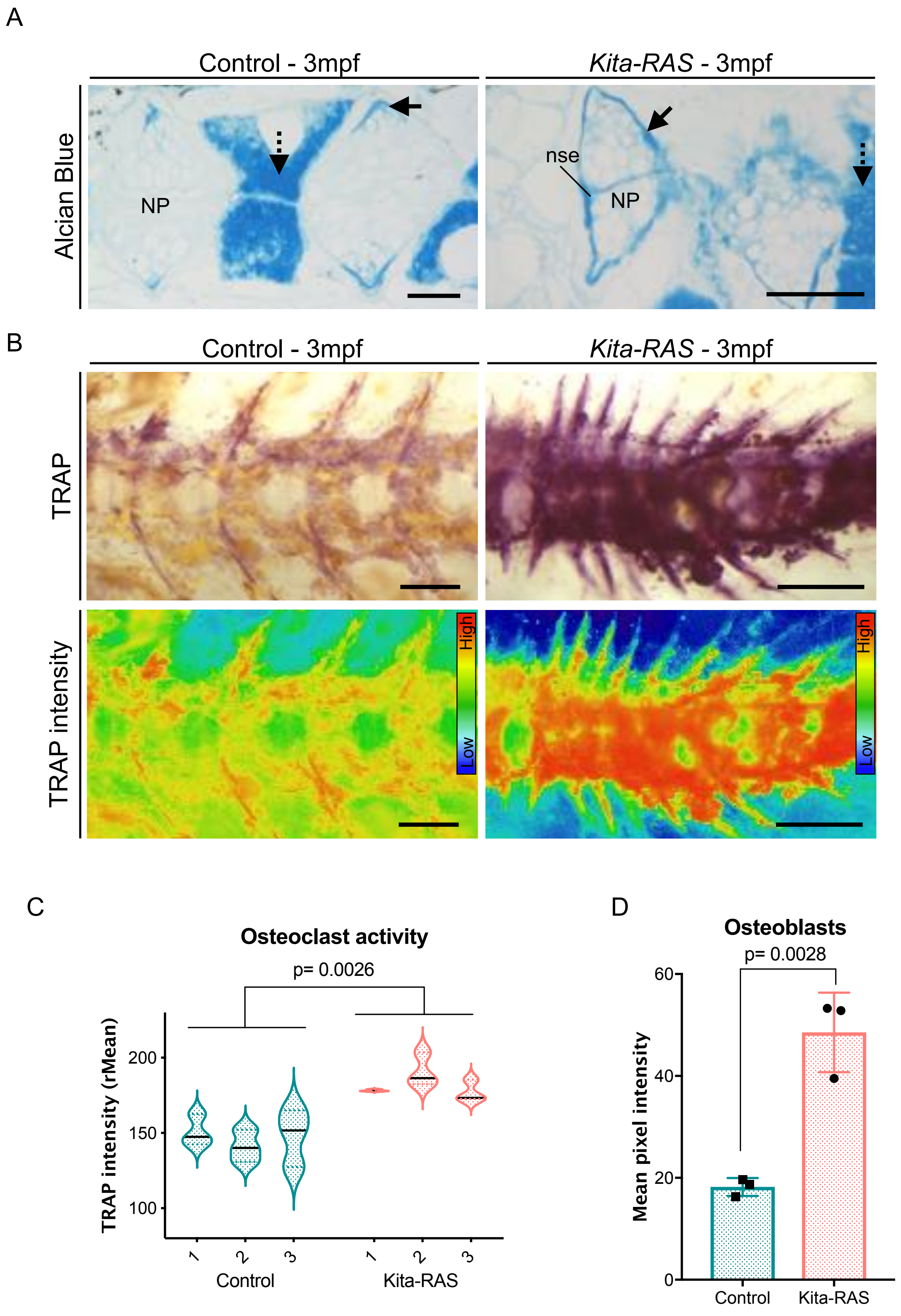
A
Control - 3mpf
Kita-RAS - 3mpf
Alcian Blue
NP
nse
NP
B
Control - 3mpf
Kita-RAS - 3mpf
TRAP
TRAP intensity
High
Low
High
Low
C
Osteoclast activity
p= 0.0026
TRAP intensity (rMean)
200
150
100
1
2
3
Control
1
2
3
Kita-RAS
D
Osteoblasts
p= 0.0028
Mean pixel intensity
60
40
20
0
Control
Kita-RAS

Figure S5. Increased glycosaminoglycans in IVDs of *Kita-RAS* and increased osteoblast and osteoclastic activity. A) Histological sections of adult controls and *Kita-RAS* fish stained with Alcian Blue to show glycosaminoglycans. Alcian Blue stains cartilage (dashed arrows), the edges of vacuolated cells in the zebrafish equivalent nucleus pulposus (NP) and the internal collagen layer of the zebrafish equivalent annulus fibrosus (arrow). In *Kita-RAS*, the Alcian Blue staining extends through the notochord sheath layer (nse). B) TRAP staining was performed in whole-mount vertebral columns of controls and *Kita-RAS*. Higher intensity of TRAP activity can be visualised in red colour when pictures were converted to represent pixel intensity (Fiji). Increased staining was observed in *Kita-RAS*. IVDs and fusions are indicated in the figure. C) Graph showing quantification of TRAP activity. Unpaired two-tailed T-test was used as statistical test (three vertebrae per fish were analysed, control n= 3, *Kita-RAS* n=3). graphs display mean with SD, p values are indicated when significant ($p<0.05$). Scale bars = 50 µm.

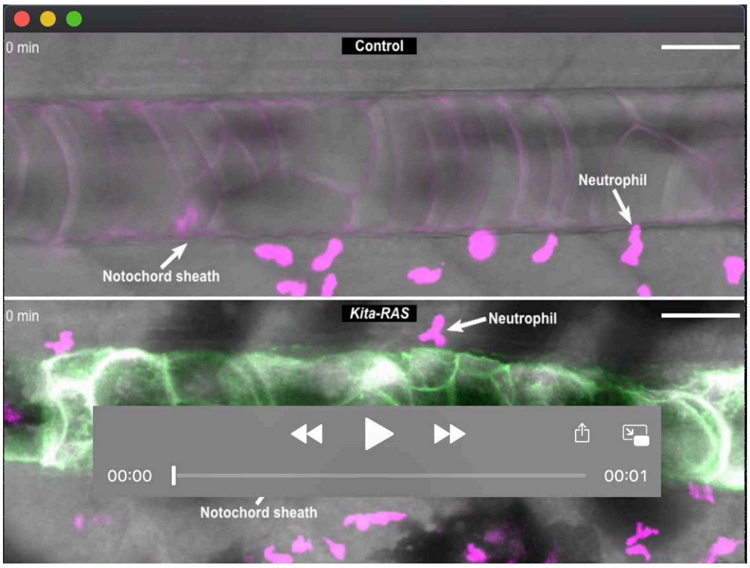

Movie 1. Neutrophils in contact with the notochord sheath in control (*Kita-mCherry*) and *Kita-RAS*. Time lapse movies of neutrophils interacting with the notochord sheath of 5dpf control and *Kita-RAS* zebrafish. Note immune cell in contact with neoplastic cells.

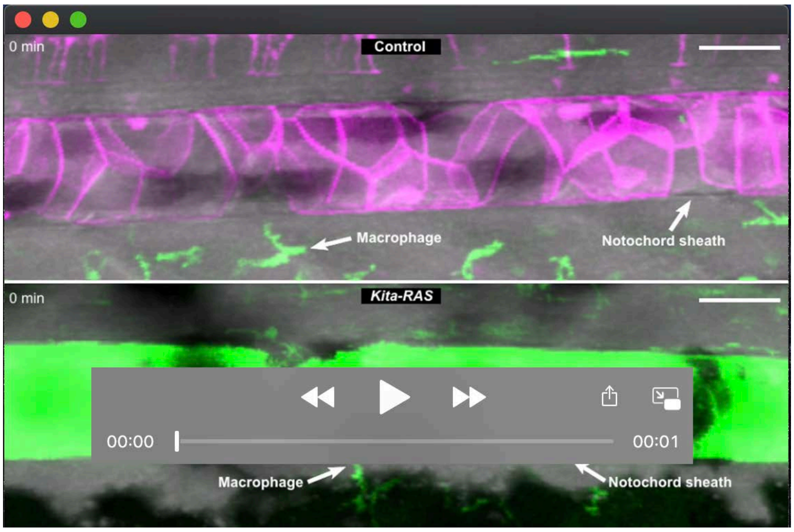

Movie 2. Macrophages in contact with the notochord sheath in control (*Kita-mCherry*) and *Kita-RAS*. Time lapse movies of macrophages interacting with the notochord sheath of 5dpf control and *Kita-RAS* zebrafish. Note immune cell in contact with neoplastic cells.
